# Predicting Synergistic and Antagonistic Micellization Effects in Surfactant Blends for Optimized Formulation Design

**DOI:** 10.1021/acs.jcim.6c02030

**Published:** 2026-09-29

**Authors:** Sofía González-Núñez, Carlos Amador, Mariano Martín

**Affiliations:** † Departamento de Ingeniería Química, 16779 Universidad de Salamanca, Pza. Caídos 1-5, 37008 Salamanca, Spain; ‡ 201091 Newcastle Innovative Centre, Procter and Gamble, Whitley Rd, Longbenton, Newcastle Upon Tyne, Tyne And Wear, NE12 9SR Newcastle upon Tyne, England

## Abstract

Surfactant mixtures exhibit non-ideal interactions that govern their macroscopic behavior, yet their prediction is limited by the need for experimentally derived interaction parameters. Within Rubingh’s Regular Solution Approximation (RST), these interactions are described by the parameter β, which is typically obtained by fitting experimental mixture data, restricting its applicability in large-scale formulation design. In this work, a computational hybrid approach combining machine learning with thermodynamic modeling is introduced to enable end-to-end prediction and optimization of surfactant mixtures. A dataset of interaction parameters derived from experimental binary surfactant mixtures was constructed to train predictive models for β, together with models for pure-component properties. The predicted, composition-dependent β values are incorporated into the RST framework to estimate mixture behavior without requiring experimental mixture data, while treating the resulting activity coefficients as effective quantities. The overall framework was further validated against a comprehensive dataset of binary surfactant mixtures and further evaluated on ternary systems, showing good agreement with experimental values across diverse interaction regimes and compositions. In addition, a multi-objective optimization strategy is introduced to identify formulations that balance performance and molecular complexity, enabling efficient exploration of chemical space. A case study on representative systems further demonstrates the applicability of the framework for computational formulation design. Overall, the proposed approach provides a scalable computational tool for surfactant mixture design, reducing reliance on experimental parameterization while enabling rapid virtual screening of formulation space.

## Graphical abstract

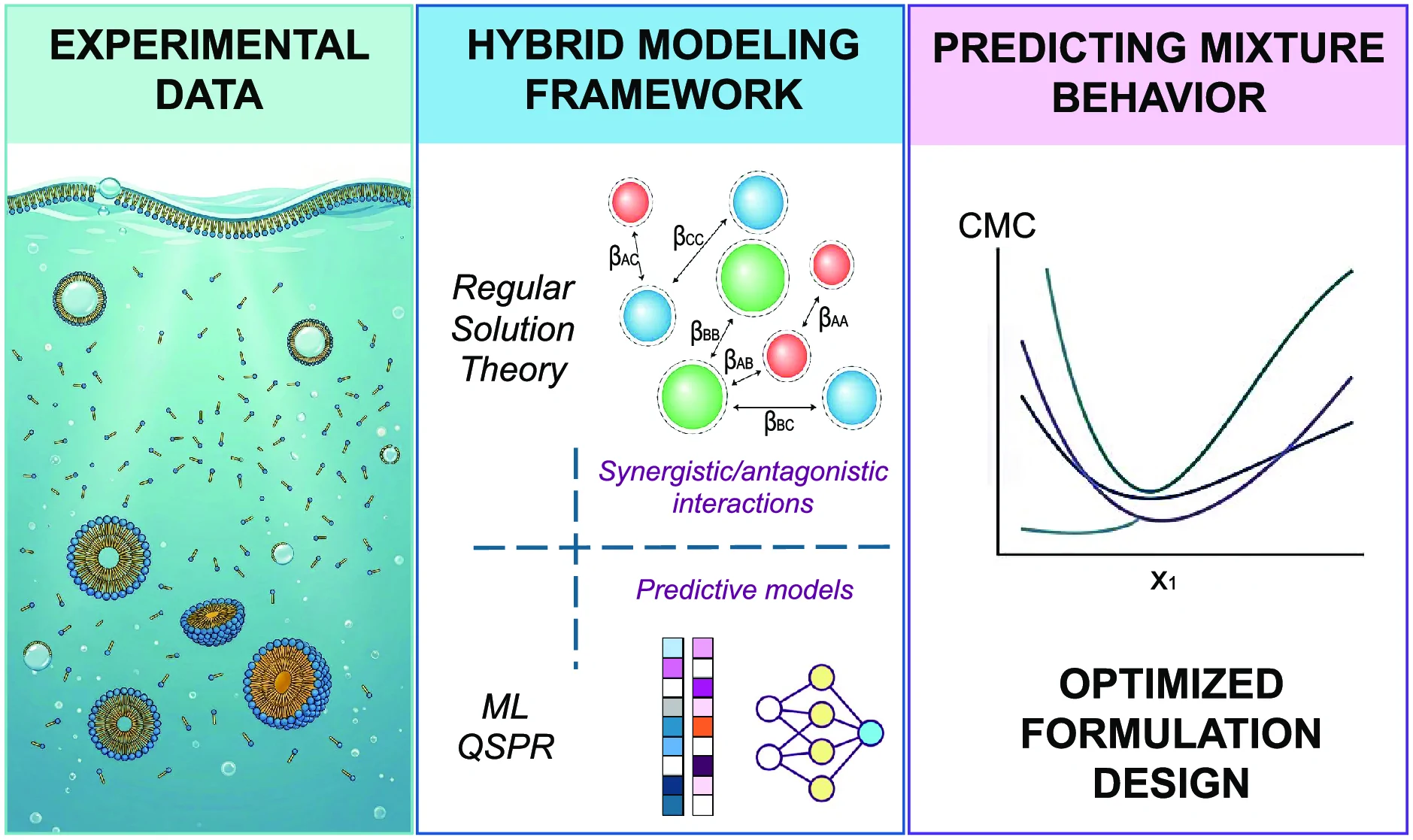

## 1 Introduction

Surfactants, short for surface-active agents, play a pivotal role in a wide array of industrial and scientific applications due to their unique ability to reduce interfacial tension and self-assemble into organized structures known as micelles. These amphiphilic molecules, characterized typically by a hydrophilic (polar) head and a hydrophobic (non-polar) tail are essential for numerous physicochemical processes including solubilization, emulsification, dispersion, and wetting.^1^ The functional versatility of surfactants underpins their widespread use across multiple sectors, ranging from detergents, personal care products, and pharmaceuticals, to food processing and petroleum recovery.^2–4^ However, in practical applications, surfactants are seldom used in isolation. Instead, formulations rely on binary or multicomponent mixtures composed of surfactants with differing ionic characteristics that allow for tuning of performance parameters to meet specific functional requirements.^5^ These combinations enable improved efficiency, reduced surfactant loading, and enhanced formulation stability. For example, mixed surfactants in detergents improve cleaning power at lower dosages, lowering both production costs and environmental impact.^6^ In pharmaceuticals, mixed micelles enhance the solubilization and bioavailability of poorly water-soluble drugs,^7^ while in cosmetics, they contribute to foam stability, mildness, and skin compatibility.^8^ Likewise, in enhanced oil recovery, tailored surfactant mixtures stabilize emulsions and optimize interfacial activity.^9^

A central phenomenon governing surfactant performance is micellization, whereby surfactant molecules aggregate in solution to form micelles once a certain concentration threshold is exceeded. This threshold, known as the critical micelle concentration (CMC), is a fundamental parameter in colloid and interface science. The CMC does not only signal the onset of micelle formation but also determines the efficiency of surfactant-based systems. While CMC depends on structural and environmental factors such as surfactant architecture, temperature, or electrolyte content, it is particularly sensitive to the presence of other surfactants. Blending surfactants with complementary properties can yield a mixture with a CMC significantly lower than that of the pure components. This non-ideal behavior arises from intermolecular interactions involving hydrophilic headgroups (e.g., electrostatic forces and hydrogen bonding), hydrophobic tail groups (e.g., van der Waals interactions), or mixed head-to-tail associations. These interactions can lead to either synergistic enhancement or antagonistic inhibition of micellization.^10^ These effects have significant practical implications. Synergistic interactions reduce CMC and improve functional properties such as solubilization capacity, foam stability, or surface tension reduction. Conversely, antagonistic interactions can hinder micelle formation or compromise system stability. Numerous experimental studies have demonstrated the advantages of mixed surfactant systems over single-component formulations, emphasizing the potential of tailored combinations for enhanced performance in real-world applications. From both theoretical and practical standpoints, these deviations from ideal mixing are crucial for formulation design, where efficacy, safety, stability, cost-efficiency, and sustainability are critical considerations. With growing consumer demand for more efficient and environmentally friendly products, the development of optimized surfactant mixtures has become increasingly relevant. By exploiting synergistic interactions, innovative formulations can achieve desired performance at lower total surfactant concentrations, aligning with the principles of green chemistry and sustainable development.^11^ Determining the CMC is often a time-consuming and costly process, typically performed using techniques such as tensiometry, refractometry, calorimetry, or measurements of viscosity and conductivity. Consequently, the prediction of surfactant properties without extensive experimentation has long been a major research goal, primarily addressed through quantitative structure–property relationship (QSPR) models. However, most of these models have been developed for individual (single-component) surfactants, limiting their applicability to practical formulations. The behavior of mixed surfactant systems cannot be straightforwardly inferred from that of their pure components, as their performance often deviates significantly from ideality due to complex intermolecular interactions. Hence, current research efforts have increasingly focused on extending predictive models to surfactant mixtures,^12^ where accurately capturing synergistic and antagonistic effects remain a major challenge. A deeper understanding of mixed micelle behavior and of the factors that govern their non-ideal interactions, is essential for advancing both the science and application of surfactants in modern industry. To interpret and quantify the behavior of mixed surfactant systems, several theoretical frameworks have been proposed, including those of Motomura,^13^ Rosen,^14^ Rubingh-Holland,^15^ Blankschtein^16^ and Maeda.^17^ Among them, Rubingh’s Regular Solution Theory (RST) has become the most widely applied due to its simplicity and ability to fit experimental data. Holland and Rubingh expanded this approach by formulating a generalized model for multicomponent non-ideal mixed micelles grounded in the pseudo-phase separation concept. In this model, the key parameter β represents the net interaction energy between different surfactant species within the micelle. The magnitude of the parameter indicates the strength of the interaction, with larger absolute values corresponding to stronger effects. A negative β value indicates favorable (synergistic) interactions, while a positive value suggests unfavorable (antagonistic) interactions. An ideal mixture, where interactions between dissimilar molecules are equivalent to those within individual species, yields β = 0. Rubingh’s model uses the experimentally determined CMC values of the pure surfactants and their mixtures to iteratively estimate the micellar mole fractions of the surfactant components. Despite certain assumptions, such as ignoring micelle shape transitions or pre-micellar aggregation, this model provides a valuable tool for studying diverse surfactant combinations. In Rubingh’s model, the interaction parameter β is assumed to be independent of composition.^18^ Although this condition is satisfied by some anionic–nonionic mixtures, in most systems β varies with composition.^19^ Notably, its evaluation relies on determining the micellar mole fraction through an iterative procedure, which is highly sensitive to variations in the CMC values of the individual surfactants. Thus, a detailed understanding of synergistic and antagonistic behavior in mixed surfactant systems is vital for designing products that fulfill demanding performance requirements across industries. Given these theoretical considerations and the limitations of existing models, numerous binary systems have been experimentally investigated to elucidate synergistic and antagonistic effects. For instance, anionic–nonionic mixtures often display pronounced synergism. The combination of sodium dodecyl sulfate (SDS) with the nonionic surfactant Triton X-100 consistently displays pronounced synergism, with reported β values of around −2.2.^20–22^ Similarly, nonionic–cationic mixtures often exhibit strong synergistic behavior. Ruiz and Aguiar^23^ reported β values of −2.6, −1.7, and −1.0 for Triton X-100 when combined with dodecyltrimethyammonium bromide (DTAB), tetradecyltrimethyammonium bromide (TTAB), cetyltrimethylammonium bromide (CTAB), respectively. While the TX100–CTAB system showed constant β across the composition range, the TX100–TTAB and TX100–DTAB mixtures exhibited slight composition dependence. Similarly, Ćirin et al.^24^ observed pronounced synergism in polysorbates Tween 20 or Tween 60 combined with sodium cholate (NaC) or sodium deoxycholate (NaDC), with the Tween 60/NaC system reaching an average β value of −7.39, compared to −3.86 for Tween 20/NaDC. These results further support that the interaction strength depends strongly on the molecular structure of both components: longer hydrophobic chains in the nonionic surfactant enhance hydrophobic interactions with the steroid rings of bile salts, while the more hydrophilic NaC favors additional hydrogen bonding, thereby amplifying the overall synergistic effect. Recent studies on mixtures of novel short-chain fluorocarbon surfactants with conventional surfactants such as CTAB have also display strong non-ideal interactions, with the maximal synergy (β = −3.662) at 0.2 mole fraction of CTAB.^25^ It is worth noting that synergism does not occur in all combinations of nonionic and ionic surfactants, particularly when dimeric (gemini) anionic and cationic surfactants are involved.^26^ On the other hand, anionic–cationic mixtures are among the most extensively studied micellar systems due to the strong electrostatic attraction between oppositely charged head groups, which contributes to the development of high-performance detergents, among others.^27^ These mixtures offer exceptional synergistic benefits, but their peak performance often coincides with phase formation (i.e., gels, coacervates, and precipitates).^28^ Recently, Li et al.^29^ investigated the interaction behavior of a sulfonic acid–based fluorinated surfactant (BQSF-SS) in mixtures with the cationic DTAB. The interaction parameter β was found to vary markedly with composition, showing a value of −12.34 at a 1:1 molar ratio (BQSF-SS:DTAB) and reaching −28.92 at 1:10. Notably, at this latter ratio, both the surface tension and the CMC attain their minimum values. Moreover, mixtures containing zwitterionic surfactants are also of particular interest because their overall neutrality at neutral pH and in the absence of added electrolytes leads to milder bio-interaction behavior, resulting in lower toxicity and higher biocompatibility. These features have promoted their growing use particularly in personal care, pharmaceutical, and agrochemical formulations. However, the strength of synergistic effects in such systems strongly depends on the cosurfactant type and the structural characteristics of the zwitterionic headgroup.^30^ These interactions are typically most pronounced with anionic surfactants, resulting in enhanced interfacial rigidity and solubilization, along with reduced surface tension and CMC compared to individual components. Mohamad-Aziz et al.^31^ investigated a binary mixture of the anionic surfactant AOT (sodium bis­(2-ethylhexyl) sulfosuccinate) and the zwitterionic SB3–12 (3-*N*,*N*-Dimethyllaurylammonio propanesulfonate), reporting a notably low β value of −21.38 at X_SB3–12_ = 0.8.

While Rubingh’s theory offers profound insights into synergistic or antagonistic effects, its practical application is resource-intensive. It requires the determination of the CMC for individual surfactants and the mixture across various compositions to calculate these interaction parameters, demanding significant time and experimental effort. These challenges have motivated the development of more efficient methodologies to capture the complex interactions governing mixed surfactant behavior. In recent years, the advent of Machine Learning (ML) has opened new avenues to tackle this challenge. Promising advancements have been recently made in predicting the CMC of binary surfactant mixtures using ML-driven approaches. Recent studies, such as those by Choudhary et al.^32^ and Qing et al.^33^ have demonstrated the capability of ML models to predict mixture CMC and surface tension, respectively, based on molecular descriptors of individual surfactants. These approaches adopt either an aggregation strategy, where component-level descriptors are averaged to represent the mixture, or a concatenation strategy, in which descriptors of all individual components, along with mixture composition, are used explicitly as model inputs. In addition, alternative representations based on graph neural networks and averaged molecular fingerprints have also been explored for this purpose, as reported by Brozos et al.^34^

However, despite these advancements, current research focuses on the direct prediction of the mixture’s CMC without explicitly addressing the underlying interaction mechanisms. While predictive, these models fail to explicitly elucidate the nature of synergistic or antagonistic interactions that are inherent to their behavior. The ability to accurately predict and interpret interaction parameters is essential for the a priori design of optimized surfactant mixtures and for advancing mechanistic understanding of their behavior. It is worth noting that deviations between ideal and experimental CMC values have been proposed as indicators for hazard assessment in mixed systems.^35^ In this context, the interaction parameter β could also provide a basis for ecotoxicity assessment, pointing to a broader applicability of predictive models that incorporate such parameters. Furthermore, multi-scale feature importance analysis identifies concentration, mixture composition, and interaction parameters as the primary determinants governing surface tension in mixed surfactant systems.^33^ All this considered, an in silico hybrid framework for the prediction and optimization of surfactant mixtures is proposed in this work. The approach combines machine learning models with a classical thermodynamic model for mixed micelle formation to enable the prediction of surfactant mixture behavior beyond direct CMC estimation strategies. Unlike traditional experimental or purely data-driven approaches, the proposed framework integrates predictive modeling with a thermodynamic framework based on RST, allowing for the rapid virtual screening of large chemical spaces. In this framework, the predicted interaction parameter is represented as a composition-dependent function, β­(x), and incorporated into the RST equations to estimate mixture behavior. Accordingly, the resulting activity coefficients should be interpreted as effective rather than strictly thermodynamically rigorous RST activity coefficients. Additionally, the formulation problem is addressed as a multi-objective optimization task, targeting both performance and synthetic feasibility.

## 2 Methodology

In this work, an in-silico hybrid framework for the prediction and optimization of surfactant mixtures is presented, moving beyond conventional direct CMC estimation approaches. The methodology integrates ML models with classical mixed micelle theory in a two-stage predictive strategy. First, a predictive model is developed to estimate the interaction parameter β, enabling the description of non-ideal interactions between surfactants. Subsequently, the predicted β values are integrated within Rubingh’s regular solution theory to estimate the CMC of unknown surfactant mixtures without requiring experimental measurements. The properties of the pure surfactants are independently predicted using ML-based models, although experimentally determined values can optionally be supplied when available. Beyond property prediction, the proposed hybrid framework enables the systematic exploration of formulation space to identify both optimal surfactant combinations and composition ratios. To this end, the problem is formulated as a multi-objective optimization task that simultaneously minimizes the mixed-micelle CMC, used as a proxy for performance, and the synthetic accessibility score (SAScore), representing molecular complexity. Importantly, once β’s are computed, the entire workflow operates without requiring experimental input data, making the approach fully predictive, scalable, and suitable for the rapid virtual screening of large chemical spaces. By coupling data-driven models with thermodynamic theory, the proposed hybrid methodology provides a general framework for the accelerated design of surfactant formulations with optimized performance–feasibility trade-offs. The overall workflow is summarized in Figure 1.

**Figure 1:**
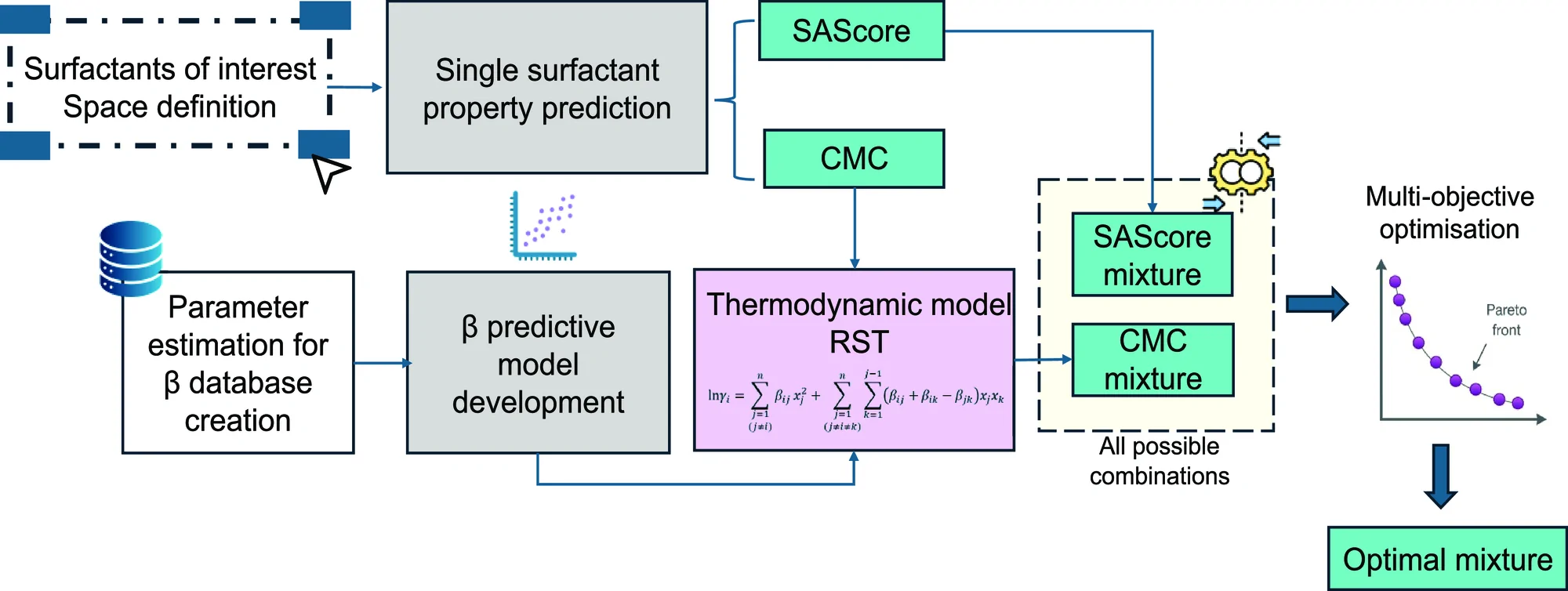
Schematic representation of the overall workflow for the hybrid in silico optimization of surfactant formulations. RST denotes Regular Solution Theory.

### 2.1 CMC Prediction for Binary Blends

This section introduces the Rubingh regular solution theory (RST) and describes the numerical estimation of its parameters from experimental data of mixtures.

#### 2.1.1 Interaction Parameters (β) in Surfactant Mixtures

The self-assembly of surfactant mixtures into micelles is governed by a complex interplay of intermolecular interactions. To quantify these interactions and predict the critical micelle concentration (CMC) of binary systems, Rubingh’s solution theory, based on the pseudo-phase separation model, was employed. Within this framework, micellization is described as an equilibrium between two pseudo-phases: a monomeric phase consisting of dispersed surfactant molecules and a micellar phase comprising aggregated species.

The fundamental condition of equilibrium requires equality of the chemical potential (μ) of each surfactant species *i*in both phases. For the monomeric phase

1 $$\mu_{i}^{monomer}=\mu_{i}^{0}+\mathrm{RT}\ln\left(X_{i}\right)$$

where μ_*i*_^0^ is the standard chemical potential, *R* is the gas constant, *T* is the absolute temperature, and *X*_*i*_ is the mole fraction of surfactant *i* in the bulk solution.

Similarly, for surfactant *i* in the micellar phase

2 $$\mu_{i}^{micelle}=\mu_{i}^{0}+\mathrm{RT}\ln\left(X_{i}^{m}\cdot f_{i}\right)$$

where *X*_*i*_^*m*^ is the mole fraction of surfactant *i* in the mixed micelle, and *f*_*i*_ is its activity coefficient in the micelle, accounting for non-ideal mixing.

At equilibrium, μ_*i*_^monomer^ = μ_*i*_^micelle^. For a pure surfactant *i* at its critical micelle concentration (CMC_*i*_), the micelle consists exclusively of that component (*X*_*i*_^*m*^ = 1, *f*_*i*_ = 1), leading to

3 $$\mu_{i}^{0}=-\mathrm{RT}\ln\left(CMC_{i}\right)$$

Combining eqs ([Disp-formula eq1])–(3) and enforcing equilibrium between phases yields the fundamental relationship given by eq ([Disp-formula eq4])

4 $$\frac{X_{i}\cdot CMC_{mix}}{X_{i}^{m}\cdot f_{i}}=CMC_{i}$$

where CMC_mix_ is the critical micelle concentration of the mixture.

To account for deviations from ideal mixing, Rubingh’s model incorporates regular solution theory, in which non-ideality is described through a single interaction parameter β. This parameter represents the net effect of intermolecular interactions between different surfactant species within the micelle. For a binary mixture (*i*/*j*), the activity coefficients are given by

5 $$f_{i}=\exp\left[\beta {\left(1-X_{i}^{m}\right)}^{2}\right]$$

6 $$f_{j}=\exp\left[\beta {\left(X_{i}^{m}\right)}^{2}\right]$$

Substituting eqs ([Disp-formula eq5]) and (6) into eq ([Disp-formula eq4]), combined with the mass balance, leads to an implicit expression for the mixed CMC

7 $$\frac{1}{CMC_{mix}}=\frac{X_{i}^{m}}{\exp\left[\beta {\left(1-X_{i}^{m}\right)}^{2}\right]\cdot CMC_{i}}+\frac{\left(1|X_{i}^{m}\right)}{\exp\left[\beta {\left(X_{i}^{m}\right)}^{2}\right]\cdot CMC_{j}}$$

Additionally, the relationship between the mole fraction of surfactant A in the mixed micelle (*X*_*i*_^*m*^) and its mole fraction in the bulk solution (α_*i*_) is derived from the equilibrium conditions and can be expressed as in eq ([Disp-formula eq8])

8 $$\frac{{\left(X_{i}^{m}\right)}^{2}\ln\left(\frac{\alpha_{i}\cdot CMC_{mix}}{X_{i}^{m}\cdot CMC_{i}}\right)}{{\left(1-X_{i}^{m}\right)}^{2}\ln\left(\frac{\left(1-\alpha_{i}\right)\cdot CMC_{mix}}{\left(1-X_{i}^{m}\right)\cdot CMC_{j}}\right)}=1$$

Equations ([Disp-formula eq7]) and ([Disp-formula eq8]) define a coupled nonlinear system in which both the micellar composition (*X*_*i*_^*m*^) and the interaction parameter (β) are unknown.

#### 2.1.2 Numerical Estimation of β

In this work, a database of interaction parameters β was generated by fitting Rubingh’s model to experimental CMC data collected from the literature for a wide range of binary surfactant mixtures.^32–34^ The resulting database was then used to develop a predictive model capable of estimating β values in previously uncharacterized mixtures, enabling the prediction of their CMCs without requiring additional experimental measurements. An important aspect of the proposed framework is the treatment of the interaction parameter β. In previous RST-based studies, β is calculated at each experimental composition and subsequently averaged to obtain a single representative value for the corresponding binary surfactant pair. For example, Qing et al.^33^ determined β at five different mixing ratios and used the arithmetic mean as the interaction parameter of the binary system. In the present work, the composition-specific β values were retained rather than replaced by a single averaged value. This choice was motivated by the structure of the dataset, in which the number of available composition points varies considerably among binary pairs, ranging from only two measurements for some systems to more than ten for others. By retaining the β value associated with each composition, the model can learn the experimentally observed dependence of the effective interaction parameter on mixture composition while making use of all available experimental observations. Accordingly, the interaction parameter is treated as a composition-dependent effective parameter, β = β­(*x*), throughout this work.

The parameters are estimated by minimizing the deviation between the experimentally measured CMCs of surfactant mixtures and those predicted by the thermodynamic model, using as inputs the reported experimental CMCs of the pure surfactants constituting each blend. This leads to the following nonlinear least-squares problem

9 $$\min_{\beta }L\left(\beta \right)=\sum_{k=1}^{N_{\exp}}{\left[{\mathrm{CMC}}_{\mathrm{mix},k}^{\mathrm{pred}}\left(\beta \right)-{\mathrm{CMC}}_{\mathrm{mix},k}^{\exp}\right]}^{2}$$

Here, CMC_mix_^pred^ is obtained by solving the coupled system of eqs ([Disp-formula eq7]) and (8), meaning that the predicted CMC depends implicitly on β through both activity coefficients and micellar composition. As a result, the estimation of β constitutes a nonlinear and strongly coupled inverse problem.

The optimization problem is solved numerically using the Broyden–Fletcher–Goldfarb–Shanno (BFGS) algorithm implemented in the Optimize module of SciPy in Python.^36^ At each iteration, the parameter vector is updated according to the quasi-Newton BFGS scheme

10 $$\beta_{k+1}=\beta_{k}-\alpha_{k}H_{k}\nabla L\left(\beta_{k}\right)$$

where ∇*L* is the gradient of the loss function (evaluated numerically), *H*_*k*_ is the inverse Hessian approximation, and α_*k*_ is the step length obtained via a line search that enforces the Wolfe conditions. The Hessian approximation is updated iteratively, enabling efficient convergence without explicit computation of second derivatives.^37^

A key feature of this approach is that each evaluation of the objective function requires solving the micellar equilibrium equations. Therefore, the estimation of β involves a nested numerical procedure in which thermodynamic consistency and agreement with experimental data are simultaneously enforced.

### 2.2 Property Prediction Models

In this study, molecular descriptors were computed using the Mordred Python library,^38^ which generates a comprehensive set of structural and physicochemical features directly from SMILES representations. These descriptors were subsequently used to develop predictive models for the interaction parameter β as well as for the properties of pure surfactants. This framework generates a comprehensive and chemically diverse descriptor space comprising 0D descriptors (e.g., atom-type counts), 1D descriptors (e.g., functional-group counts), and 2D descriptors (e.g., connectivity-based topological indices), yielding more than 1600 molecular descriptors for each molecule. Given the high dimensionality of the resulting feature space, a systematic feature selection procedure was applied to ensure model robustness and statistical tractability. First, descriptors containing missing values, non-numeric entries, or constant values across the dataset were removed. Subsequently, multicollinearity was addressed by computing pairwise Pearson correlation coefficients across all descriptors. Highly correlated features (|*r*| > 0.9) were eliminated, ensuring that only one representative descriptor is retained from each highly correlated group. This step reduces redundancy and improves model stability by avoiding overlapping information across inputs. Because this filtering procedure depends only on inter-descriptor correlations and not on the response variable, it constitutes an unsupervised preprocessing step and is not expected to introduce optimistic bias associated with supervised feature selection. After preprocessing, the resulting descriptor set was significantly reduced while preserving chemical diversity and informational content.

#### 2.2.1 Pure-Component Property Prediction

Machine learning models were developed to estimate the pure-component CMC of individual surfactants, together with the intermolecular interaction parameter (β) governing non-ideal mixing effects in binary surfactant systems. The predicted pure-component properties and interaction parameters were subsequently integrated within the Rubingh’s Regular Solution Theory (RST) to estimate the CMC of surfactant mixtures. Rather than directly estimate mixture CMC values through a purely data-driven correlation, the proposed hybrid methodology combines machine learning with thermodynamic theory to interpret the physicochemical relationships underlying mixed micellization. This strategy preserves the interpretability and physical consistency of thermodynamic modeling while maintaining the predictive flexibility of machine learning approaches

To extend the framework toward molecular design applications, the Synthetic Accessibility Score (SAScore) was included as an additional optimization objective. The SAScore is a widely used heuristic molecular descriptor that estimates the synthetic feasibility of a compound directly from its chemical structure. It is based on fragment contributions and global structural complexity penalties, including factors such as uncommon functional groups, stereochemical complexity, macrocycles, branching, and scaffold topology. The resulting score is defined on a relative scale from 0 to 10, where higher values indicate molecules that are potentially more challenging to synthesize. In the present framework, the SAScore of a surfactant mixture was calculated as the mole-fraction-weighted average of the SAScore values of the pure surfactants. This descriptor was incorporated as a secondary objective alongside CMC minimization, enabling the prioritization of formulations that combine favorable predicted performance with relatively lower synthetic complexity. Such a criterion is particularly useful during virtual screening, where a large number of candidate mixtures must be ranked efficiently. It should be emphasized that SAScore is used here only as a comparative ranking descriptor and not as a rigorous measure of synthetic feasibility. Because it is derived from fragment statistics and structural complexity heuristics, SAScore does not account for actual reaction pathways, route availability, process complexity, or the accessibility of novel molecular fragments. Accordingly, the SAScore-based objective should be interpreted as a practical surrogate for relative synthetic complexity rather than a guarantee of experimental synthesizability.

#### 2.2.2 β Prediction Model

Alternative descriptor aggregation schemes are evaluated to construct the input representation of binary surfactant mixtures, each reflecting a different assumption about how molecular information and composition jointly determine mixture behavior. The first approach, the concatenation strategy, preserves the full identity of both components by explicitly retaining their individual descriptor vectors together with their corresponding mole fractions. The mixture is therefore represented as

11 $$X=\left[X_{i}\left|\right|X_{j}\left|\right|x_{i}\left|\right|x_{j}\right]$$

where *X*_1_ and *X*_2_ denote the molecular descriptor vectors of each surfactant and *x*_1_, *x*_2_ their mole fractions.

This representation allows the learning model to independently access component-specific information while explicitly incorporating composition.

The second scheme, the arithmetic mean strategy, adopts a fully aggregated representation based on a linear mixing assumption

12 $$X=x_{i}X_{i}+x_{j}X_{j}$$

which implicitly assumes that molecular contributions to the mixture properties are linearly additive in descriptor space.

Finally, the harmonic mean strategy introduces a nonlinear aggregation scheme designed to capture inverse-type interactions between components. In this case, the mixture representation is defined as follows:

13 $$X=\frac{1}{\frac{x_{i}}{X_{i}}+\frac{x_{j}}{X_{j}}}$$

where the operation is applied element-wise over the descriptor vectors. This formulation potentially captures non-additive mixing behavior that is not well represented under linear assumptions.

### 2.3 Binary Blend Optimisation Pipeline

The predictive framework is embedded within a combinatorial screening pipeline for surfactant mixtures. A library of candidate surfactants is defined using SMILES representations, from which molecular descriptors are computed using the defined descriptor set. For each unique binary pair and across a predefined mole-fraction grid, the pipeline sequentially (i) computes molecular descriptors, (ii) predicts the interaction parameter β, (iii) estimates pure-component CMC values, and (iv) evaluates the mixture CMC using Rubingh’s regular solution theory. Nonetheless, practical formulation design involves competing objectives, i.e., achieving the lowest possible CMC may conflict with using molecules that are synthetically accessible or commercially available. The multi-objective optimisation proposed addresses this conflict by computing Pareto-optimal trade-off fronts between any user-defined set of mixture objectives.

#### 2.3.1 Epsilon-Constraint Method

The ε-constraint method is a practical approach for solving multi-objective optimization problems by converting them into a sequence of single-objective subproblems.^39^ The key idea is to maximize or minimize one selected objective function while the remaining objectives are incorporated as constraints bounded by ε parameters. By systematically varying these ε values, an approximation of the Pareto front can be obtained. In this formulation, one objective function *f*_1_(*y*) is chosen as the primary objective and minimized over the feasible region *S*, while the other objectives are treated as constraints. For problems with more than two objectives, each additional objective introduces a separate ε-constrained inequality. Considering all objectives are minimized, the model is expressed as:

$$\underset{y\in S}{\min}g_{1}\left(y\right)$$

subject to

14 $$g_{i}\left(y\right)\leq \varepsilon_{i},i=2,...,p$$

To properly define the ε parameters, it is first necessary to determine the range of each constrained objective function. This range is computed as follows:

15 $$r_{i}=g_{i}^{\max}-g_{i}^{\min},i=2,...,p$$

Each range is then discretized into *q*_*i*_ equal intervals, generating *q*_*i*_ + 1 grid points per objective. The total number of resulting subproblems is therefore given by ∏_*i*=2_^*p*^(*q*_*i*_ + 1). The ε values at each grid point are defined as follows:

16 $$\varepsilon_{i}^{k}=g_{i}^{\min}+k\cdot \frac{r_{i}}{q_{i}},k=0,...,q_{i}$$

A finer discretization improves the resolution of the resulting Pareto front but increases the computational cost accordingly. Hence, selecting an appropriate balance between accuracy and efficiency is essential.

### 2.4 Extension to Multicomponent Blends via Pairwise Interaction Decomposition

Predictive models for surfactant mixture properties are typically trained on binary systems due to data scarcity and the combinatorial complexity associated with ternary and higher-order mixtures.^40^ However, practical industrial formulations are inherently multicomponent in nature. The proposed framework provides a theoretically consistent route to extend binary blends to multicomponent systems by leveraging the pairwise-additive structure embedded in Rubingh’s Regular Solution Theory (RST).

#### 2.4.1 The Pairwise-Additive Nature of RST

Within the RST, the non-ideality of multicomponent micellar systems can be described in terms of composition-dependent activity coefficients expressed as functions of micellar mole fractions and pairwise interaction parameters β_*ij*_. Several studies have applied the extended RST to predict partition coefficients in ternary surfactant systems, using experimental data obtained from binary surfactant mixtures.^41–43^ For an *n*-component system, the logarithm of the activity coefficient of component *i* can be written as follows:

17 $$\ln f_{i}=\left[\sum_{\begin{array}{l} j=1\\ j\neq i \end{array}}^{n}\beta_{\mathrm{ij}}x_{j}^{2}+\sum_{\begin{array}{l} j\neq i\\ j\neq k\neq i \end{array}}^{n}\sum_{k=1}^{j-1}\left(\beta_{\mathrm{ij}}+\beta_{\mathrm{ik}}-\beta_{\mathrm{jk}}\right)x_{j}x_{k}\right]$$

Within this formalism, the thermodynamic behavior of the mixture is fully determined by the set of pairwise interaction parameters. For an *n*-component system, the total number of independent binary interaction parameters is given by the combinatorial expression $C_{2}^{n}=\frac{n!}{\left[2!\left(n-2\right)!\right]}$

A key implication of this theory is that the macroscopic thermodynamics of complex multicomponent micelles can, in principle, be reduced to the prediction of pairwise interactions. Once the activity coefficients *f*_*i*_ are determined, the mixed micellar CMC can be expressed as follows:

18 $$\frac{1}{CMC_{mix}}=\sum_{i=1}^{n}\frac{\alpha_{i}}{f_{i}CMC_{i}}$$

being α_*i*_ represents the bulk mole fraction of component *i*.

From this perspective, the predictive model developed in this work to accurately estimate β_*ij*_ enables the extension of CMC predictions beyond binary systems. In this way, micellization behavior in multicomponent surfactant mixtures can be systematically estimated.

#### 2.4.2 Decomposition of Multicomponent Into Binary Pairs

Given an N-component mixture with global mole fractions {*x*_*i*_}_*i*=1_^*N*^, define for each pair (*i*,*j*) with *i* < *j* the locally normalized fractions

19 $$x_{i}^{\left(\mathrm{ij}\right)}=\frac{x_{i}}{x_{i}+x_{j}},x_{j}^{\left(\mathrm{ij}\right)}=\frac{x_{j}}{x_{i}+x_{j}}$$

These satisfy *x*_*i*_^(*ij*)^ + *x*_*j*_^(*ij*)^ = 1, constituting a proper binary composition. Crucially, these local fractions preserve the relative stoichiometry of the (*i*,*j*) pair within the full mixture.

#### 2.4.3 Pairwise β Prediction

Let *X*_*i*_ denote the molecular descriptors of component *i*, encoding its structural and physicochemical properties. The pairwise interaction parameter is then estimated using a trained predictive model as

20 $$\beta_{\mathrm{ij}}=f\left(X_{i},X_{j},x_{i}^{\left(\mathrm{ij}\right)},x_{j}^{\left(\mathrm{ij}\right)}\right)$$

where *f* represents the predictive model mapping molecular structure and local compositional context to the effective interaction parameter estimation.

## 3 Results and Discussion

### 3.1 Pure-Component Property Prediction Models

The performance of the predictive models developed for pure-component properties as a function of Mordred molecular descriptors is summarized here. The pure-component estimation models were adopted from our previous work.^44^

- log­(CMC): the model to predict critical micelle concentration is built with data for 353 surfactants, combining data published by Qin et al.,^45^ Rosen & Kunjappu^1^ and Seddon et al.^46^ The number of descriptors decreases to 223 after filtering. PLS regression is applied with 7 PCs.
- SAscore: A database containing 269 surfactants is constructed from the model developed by Ertl and Schuffenhauer.^47^ Descriptor filtering reveals that 221 Mordred descriptors are required. PLS regression is applied with 4 principal components (PCs).

The performance metrics for both models are reported in Table 1 and parity plots shown in Figure 2.

**Figure 2:**
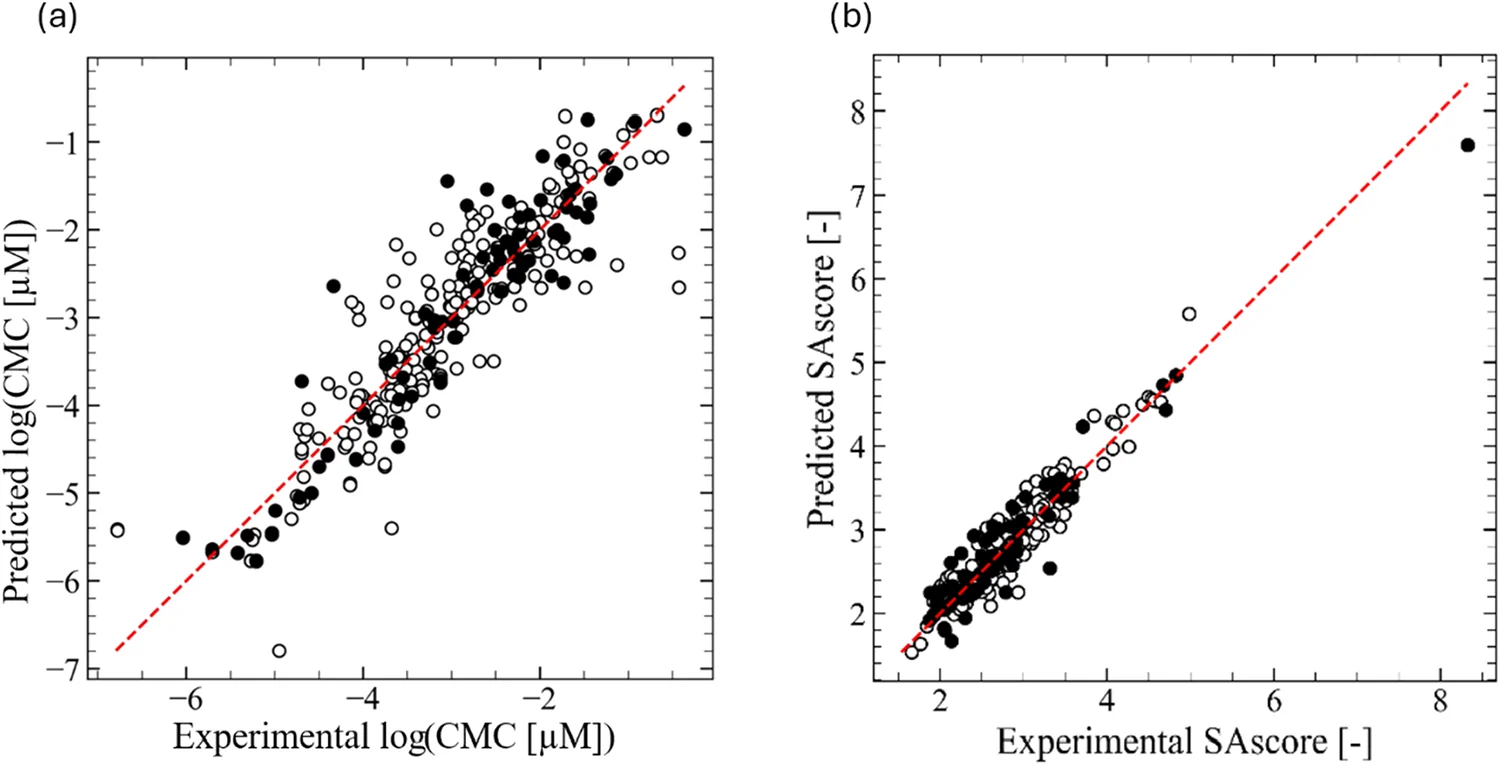
Parity plots comparing predicted and experimental values of (a) log­(CMC), where CMC is expressed in μM, and (b) Synthetic Accessibility Score. Hollow circles represent the training set, while filled circles correspond to the test set.

**Table 1:** Metrics for the Property Prediction Models^a^

Model	Units	Size of dataset	Model type	RMSE_train_	*R*_train_^2^	RMSE_test_	*R*_test_^2^
SAscore	-	269	PLS	0.24	0.90	0.21	0.91
log(CMC [μM])	–	353	PLS	0.47	0.86	0.47	0.87

a The size of the dataset denotes the total number of training and testing data. RMSE_*x*_ denotes the root mean squared error for the training (*x* = train) and testing (*x* = test) sets and carries the same units as the property of interest. Similarly, *R*_*x*_^2^ denotes the coefficient of determination for the training and testing sets, and it is dimensionless.

### 3.2 β Predictive Model

Among the descriptor aggregation strategies evaluated, the concatenation-based representation yielded the best predictive performance. Because descriptor concatenation is inherently order-dependent, we adopted a canonical pair representation combined with permutation augmentation during model training. Each binary surfactant pair was assigned a unique canonical ordering, so that pairs (*i*, *j*) and (*j*, *i*) were treated as the same physical mixture. This canonical representation was used to identify unique mixtures, construct pair-grouped cross-validation folds, and evaluate model performance. During training, each mixture was augmented by including both descriptor concatenation orders [ *X*_*i*_ || *X*_*j*_ || *x*_*i*_ || *x*_*j*_ ], [ *X*_*j*_ || *X*_*i*_ || *x*_*j*_ || *x*_*i*_ ] while retaining the same target value (β). Both augmented representations were always assigned to the same training/test partition and cross-validation fold to prevent information leakage. During evaluation, predictions were generated only for the canonical representation of each mixture, ensuring that each experimental binary mixture contributed a single prediction and target value to the reported performance metrics.

The predictive model was developed using a curated dataset comprising 688 pair-composition observations of the interaction parameter β, derived from 154 unique binary surfactant pairs formed from 106 individual surfactants. Each binary pair was measured at multiple compositions (median = 4), providing broad coverage across the composition range. The dataset spans a diverse charge space, with the strongest representation in anionic–nonionic and cationic–nonionic systems (*n* = 136 each), followed by cationic–cationic (*n* = 129), anionic–cationic (*n* = 117), anionic–anionic (*n* = 68), nonionic–nonionic (*n* = 40), nonionic–zwitterionic (*n* = 24), anionic–zwitterionic (*n* = 20), and cationic–zwitterionic (*n* = 18). Overall, the β target exhibits wide non-ideality coverage, encompassing both strong synergistic and antagonistic regimes. Such broad compositional diversity is critical for capturing the range of behaviors observed in practical surfactant formulations. Furthermore, the inclusion of Gemini surfactants further expands the structural and physicochemical diversity of the dataset, enabling the model to learn interaction patterns associated with highly surface-active and structurally complex amphiphiles. All interaction parameters were estimated from experimental CMC data of binary surfactant mixtures reported in the datasets compiled by Qing et al.,^33^ Brozos et al.,^34^ and Choudhary et al.^32^ To minimize variability arising from experimental conditions, all CMC and β data included in the present study were restricted to measurements performed at 25 °C. Literature sources reporting measurements at multiple temperatures were filtered to retain only the 25 °C data. The compiled dataset includes values obtained using different experimental techniques (e.g., surface tension, conductivity, and isothermal titration calorimetry), which were retained when measured under these conditions and treated as part of the experimental variability of the dataset. Measurements involving added electrolytes, co-solvents, or other formulation additives were excluded. Each binary mixture was represented using molecular descriptors computed with Mordred. For each individual surfactant, descriptor filtering reveals that 176 Mordred descriptors are required. To evaluate model generalization, the dataset was assessed using two complementary data-splitting strategies. In the random composition split, individual observations were randomly assigned to the training (80%) or test (20%) partitions, allowing the same binary surfactant pair to appear in both partitions at different composition points. This strategy primarily evaluates interpolation performance within the range of surfactant combinations represented in the training data. In the unseen-pair split, partitioning was performed at the level of binary surfactant pairs, with 80% of the unique pairs assigned to the training set and the remaining 20% reserved for testing, ensuring that no surfactant pair, and therefore none of its composition points, was observed during model fitting. This strategy evaluates extrapolation to previously unseen binary combinations and was considered the most relevant benchmark for the intended application of the model. Both strategies were evaluated using 5-fold cross-validation, and predictive performance is reported as the mean ± standard deviation of *R* ^2^ and RMSE across the five folds.

Three non-linear regression models were benchmarked: a multilayer artificial neural network (ANN), XGBoost, and LightGBM. Hyperparameters were optimized using Bayesian optimization within a nested cross-validation framework, in which tuning was performed exclusively on the training data of each outer fold while preserving the same grouping structure as the corresponding split, thereby preventing information leakage during model selection. Predictive performance was evaluated using *R* ^2^ and RMSE on both the training and test partitions of each fold, and the cross-validated performance of the three models under both splitting strategies is summarized in Table 2. Model selection was based primarily on performance under the unseen-pair split, where the ANN achieved the highest cross-validated *R* ^2^ and the smallest difference between training and test performance, although the performance of the three models showed substantial overlap across folds. The parity plot of the selected ANN model is shown in Figure 3, while the corresponding parity plots for LightGBM and XGBoost are provided in the Supporting Information (Figures S1–S2). After completion of the cross-validation procedure, a separate Bayesian optimization was performed using the complete dataset to obtain a final hyperparameter configuration for each model type. These final configurations, provided in Tables S1–S3, were subsequently used to train the final models on the complete dataset, which are shared in the GitHub repository. Importantly, these final configurations were not used in the cross-validation or in the end-to-end evaluation of the binary mixtures described in Section 3.3. Instead, the end-to-end evaluation used the corresponding model from each outer fold, thereby preserving the out-of-fold validation procedure. The final model is used for the prediction of ternary mixtures and for the case study on formulation optimization.

**Figure 3:**
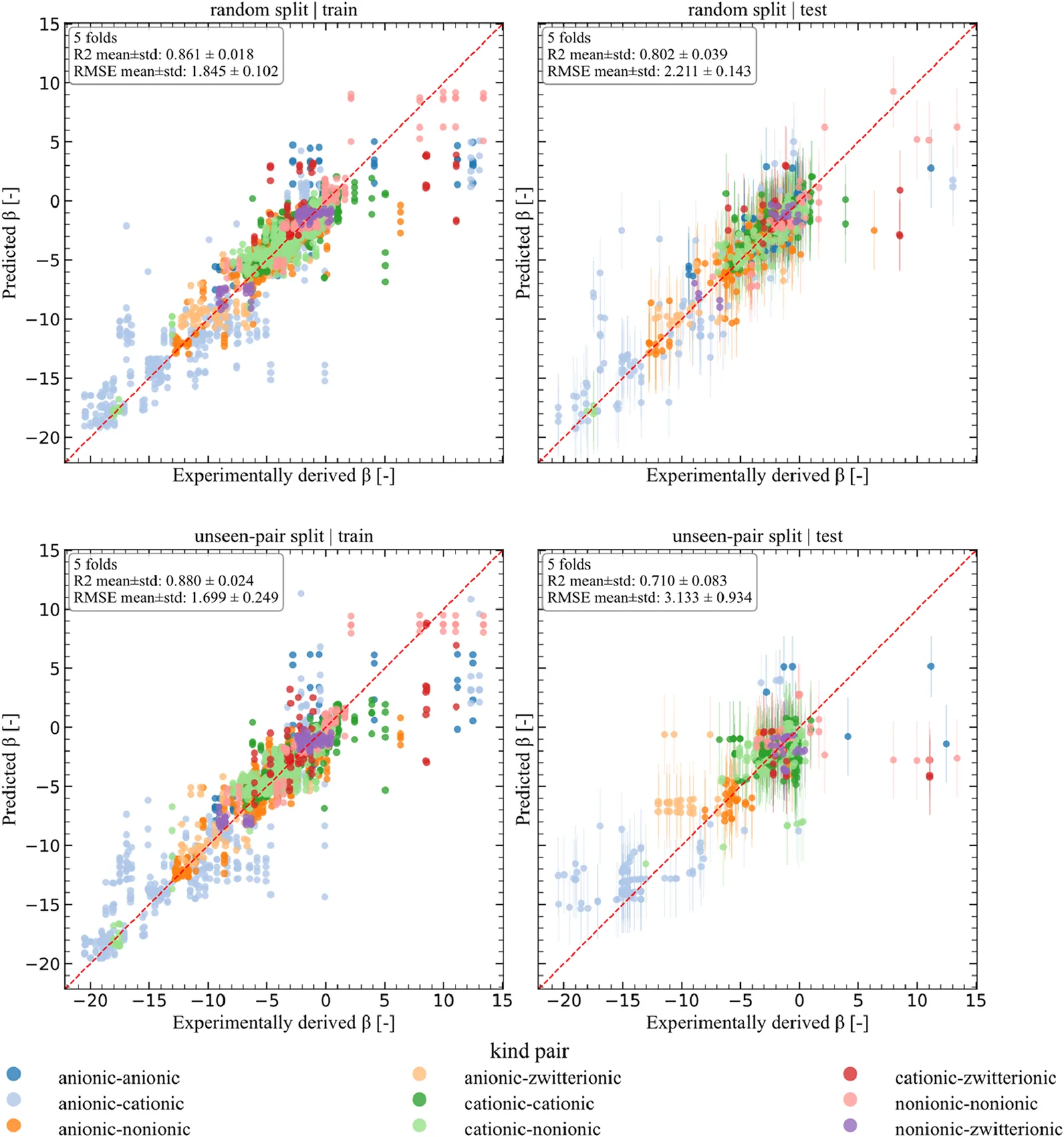
Parity plots of predicted versus experimentally derived β values for the ANN model under two data-splitting strategies. Rows correspond to the random composition split (80/20 split of individual observations; top) and the unseen-pair split (80/20 split of unique binary surfactant pairs; bottom), while columns show predictions for the training (left) and test (right) partitions. Points are coloured according to the surfactant charge-class combination (see legend). The dashed red line denotes the ideal *y* = *x* relationship. Vertical bars in the test panels represent 95% split-conformal prediction intervals.

**Table 2:** Predictive Performance of the ANN, LightGBM, and XGBoost Models under Two Data-Splitting Strategies: Random Composition Split and Unseen-Pair Split^a^

Split	Metric		ANN	LightGBM	XGBoost
Random split	Train	*R* ^2^	0.844 ± 0.014	0.882 ± 0.004	0.906 ± 0.003
		RMSE	1.975 ± 0.077	1.724 ± 0.037	1.545 ± 0.034
	Test	*R* ^2^	0.802 ± 0.039	0.825 ± 0.034	0.826 ± 0.043
		RMSE	2.211 ± 0.143	2.050 ± 0.171	1.995 ± 0.160
Unseen-pair split	Train	*R* ^2^	0.857 ± 0.013	0.888 ± 0.009	0.914 ± 0.010
		RMSE	1.835 ± 0.136	1.646 ± 0.070	1.443 ± 0.071
	Test	*R* ^2^	0.710 ± 0.083	0.685 ± 0.095	0.683 ± 0.119
		RMSE	3.133 ± 0.934	3.098 ± 0.714	3.060 ± 0.82

a The coefficient of determination (*R* ^2^) and root mean square error (RMSE) are reported for both the training and test sets as the mean ± standard deviation across five cross-validation folds.

As shown in the parity plot, the model reproduces the experimentally derived β values with good accuracy across a chemically diverse dataset comprising surfactants with different headgroup charges and molecular architectures. Data points are color-coded according to the charge class of the surfactant pair, allowing direct visualization of the synergistic (β < 0) and antagonistic (β > 0) regimes across the different chemical families represented in the dataset. Antagonistic cases are strongly underrepresented, with more than 90% of the mixtures exhibiting synergistic interactions. This imbalance is not random but chemically structured: antagonism occurs predominantly in nonionic–nonionic, cationic–cationic, and anionic–anionic pairs, whereas opposite-charge systems are almost entirely synergistic. This distribution is consistent with the expected electrostatic interactions between surfactant headgroups. Consequently, while the model correctly captures the sign and general magnitude of β across all charge classes, the statistical confidence in the antagonistic regime is inherently lower because of the limited number of available samples. This imbalance reflects the available experimental space rather than a limitation of the model formulation. Deviations become more pronounced at the extremes of the β distribution, where data density is lowest, and are therefore consistent with reduced local support in the training domain rather than systematic extrapolation bias. Importantly, no evidence of systematic failure is observed, and the predictions remain physically consistent across the full range of β values. Overall, the agreement between predicted and experimental values is robust, and the level of accuracy is sufficient to preserve the thermodynamic consistency required for subsequent mixture calculations.

All trained models used in this study, including the pure-component CMC and SAScore predictors and the ANN, XGB, and LGB models for the β-interaction parameter, are publicly available in the GitHub repository associated with this work.

### 3.3 CMC End-to-End Validation in Binary Mixtures

To evaluate the overall predictive capability of the proposed framework, mixture CMC values obtained from predicted pure-surfactant CMCs and predicted interaction parameters were compared with the corresponding experimental mixture CMC values. To ensure that this validation is free from information leakage, an independent end-to-end evaluation was performed using the unseen-pair protocol described in Section 3.2. For each of the five folds, the ANN β model was retrained from scratch using only the training pairs assigned to that fold, while the held-out binary pairs were reserved exclusively for testing. The fold-specific β predictions were then combined with the independently trained pure-component CMC model and propagated through the Rubingh regular-solution equations at the experimental composition to predict the CMC of the corresponding binary mixture. Consequently, each reported prediction was generated by a β model that had never been trained on the corresponding binary pair.

The resulting parity plot is shown in Figure 4. Across the 96 held-out binary mixtures, the end-to-end validation achieved *R* ^2^ = 0.768 and RMSE = 0.349 demonstrating that the proposed framework retains good predictive accuracy under a fully independent evaluation protocol. The complete set of predicted and experimental values for all 96 binary mixtures is available in the GitHub repository associated with this work.

**Figure 4:**
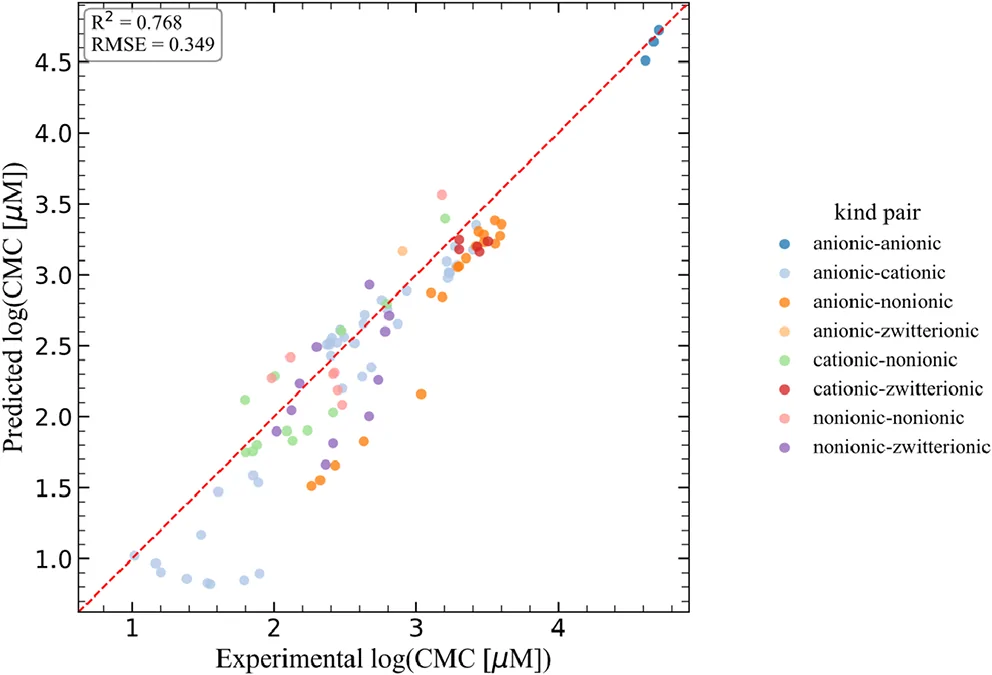
End-to-end held-out parity plot of predicted versus experimental log­(CMC [μM]) for binary surfactant mixtures. Each point represents a binary mixture whose surfactant pair was completely excluded from the training set of the β model under the unseen-pair split strategy. Points are color-coded according to the surfactant charge-pair class.

Experimental and predicted CMC profiles were compared for all held-out binary pairs containing at least four measured composition points. The model reproduces the overall CMC–composition trends and shows no systematic displacement of the CMC minimum across the major charge-pair classes represented in the dataset (Figure S3). In addition, to quantify the contribution of the interaction parameter, the end-to-end validation was repeated under the ideal-mixing assumption (β = 0) using the same 96 held-out binary mixtures. The comparison, provided in Table S4 and Figure S4, shows that incorporating the ANN-predicted β values substantially improves mixture CMC prediction accuracy.

### 3.4 CMC End-to-End Validation in Ternary Mixtures

To evaluate the applicability of the proposed framework to multicomponent systems, CMC predictions were performed for 44 ternary surfactant mixtures collected from the literature.^40,43,48–51^ The analysis covered 16 distinct ternary chemical families and 18 unique surfactants, providing broad structural and compositional diversity. A broad compositional domain was investigated, enabling the evaluation of the framework under both component-dominated and compositionally balanced ternary conditions. The evaluated systems included anionic, cationic, and nonionic surfactants, enabling the assessment of the framework across different electrostatic interaction environments. Among the unique surfactants considered, 8 were cationic, 6 anionic, and 4 nonionic. The analyzed ternary formulations comprised 132 individual surfactant occurrences, dominated by cationic species (67), followed by anionic (35) and nonionic (30) surfactants. The ternary mixtures exhibited substantial chemical heterogeneity, including cationic-only systems (19/44), anionic-only systems (2/44), anionic–anionic–nonionic mixtures (13/44), cationic–nonionic–nonionic mixtures (7/44), and fully mixed anionic–cationic–nonionic systems (3/44). This diversity ensured that the framework was evaluated under both single-charge and mixed-charge interaction regimes, where synergistic and antagonistic effects are expected to differ significantly. Of the 16 unique ternary systems, only 3 (18.8%) have all three constituent binary sub-pairs (AB, AC, and BC) represented in the binary β-model training dataset, whereas 5 systems (31.2%) contain no constituent sub-pairs present in the training set. Overall, 20 of the 28 distinct constituent binary sub-pairs (71.4%) and 9 of the 18 unique surfactants (50%), including all four long-chain PEG nonionic surfactants, are absent from the binary training dataset. This demonstrates that the ternary evaluation includes a substantial number of previously unseen surfactants and pairwise combinations, and that the model generalizes primarily through molecular and charge-class descriptors rather than memorization of specific binary pairs. Consistent with this, the predicted ternary CMC values showed good agreement with experimental data, supporting the ability of the proposed methodology to generalize from binary to multicomponent surfactant systems. The framework is therefore suitable for the ranking and virtual screening of ternary surfactant formulations in logCMC space, as illustrated in Figure 5. The complete set of predicted and experimental values for all ternary mixtures is available in the GitHub repository associated with this work.

**Figure 5:**
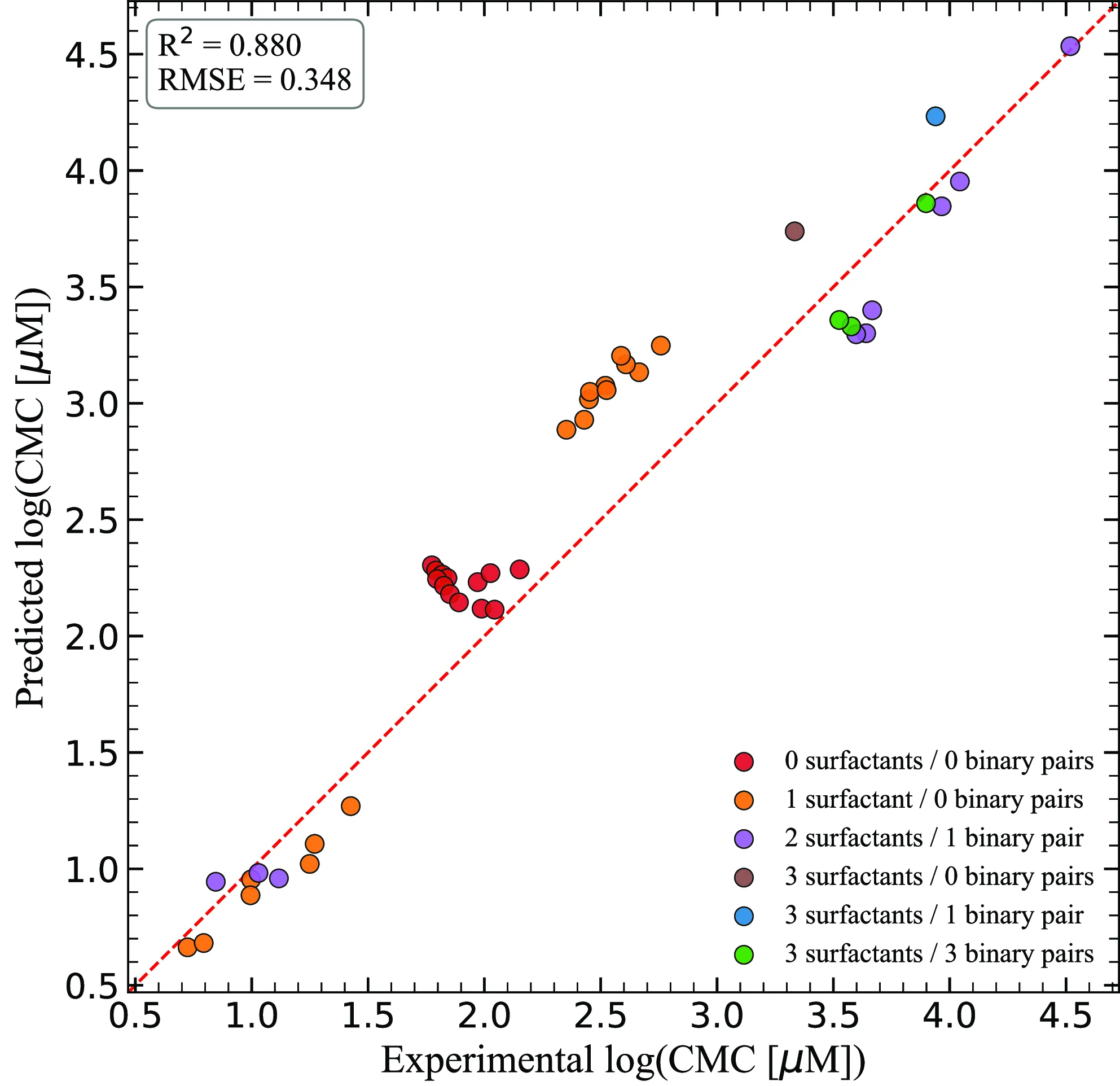
Parity plot of predicted versus experimental log­(CMC) values for ternary surfactant mixtures, with CMC expressed in μM. Points are colour-coded according to the degree of overlap between each ternary system and the binary training set. Labels of the form N surfactants / M binary pairs indicate the number of constituent surfactants and binary sub-pairs that were represented in the binary training set used to fit the ANN interaction-parameter model.

### 3.5 Case Study

The defined dataset comprises 20 representative surfactants spanning anionic, cationic, nonionic, and zwitterionic classes, selected to cover a broad physicochemical space in terms of headgroup chemistry, hydrophobic architecture, and degree of ethoxylation. The anionic subset includes lauryl sulfate (SLS), linear alkylbenzene sulfonate (LAS), ethoxylated sulfate, ethoxylated phosphate surfactants, dioctyl sulfosuccinate, and palmitoyl isethionate. Cationic systems include benzyl dimethyl tetradecyl ammonium, dodecyltrimethylammonium (DTAB), cetyltrimethylammonium (CTAB), and related quaternary ammonium surfactants. Nonionic surfactants comprise C10–C14 ethoxylates, octylphenol ethoxylate (Triton-type), alkyl polyglucosides including octyl and decyl glucoside, and a decyl maltoside-based structure. Zwitterionic species include betaine-type and sulfobetaine-type surfactants, as well as amine oxide derivatives. This selection enables the exploration of distinct intermolecular interaction regimes governing mixed-micelle formation, including electrostatic interactions, hydrogen bonding, steric hydration, and hydrophobic packing effects. The complete list of the 20 surfactants and their SMILES representations is provided in the Supporting Information (Table S5).

A total of 1900 binary surfactant blends were computationally generated by enumerating all unique pairwise combinations of the 20 selected surfactants (190 pairs) and sampling each pair across a 10-point composition grid. This procedure defines the complete combinatorial chemical space explored in this work. For each surfactant, molecular descriptors were first used to predict single-component properties along with binary β-interaction parameters, enabling the quantification of non-ideal mixing effects in surfactant pairs. The mixed-micelle CMC at each composition was subsequently computed following RST, resulting in a hybrid end-to-end pipeline that integrates data-driven predictions with classical thermodynamic theory. The design problem was formulated as a multi-objective minimization targeting both mixed-micelle CMC and SAScore, thereby capturing the inherent trade-off between physicochemical performance and synthetic accessibility. Multi-objective optimization was then performed to identify the Pareto-optimal set of non-dominated solutions. A deterministic ranking of the Pareto set was subsequently carried out using the Technique for Order Preference by Similarity to Ideal Solution (TOPSIS).^52^ TOPSIS evaluates each candidate by computing its Euclidean distance to an ideal solution (representing the best values across all objectives) and a negative-ideal solution (representing the worst values), both defined in the normalized objective space.

To evaluate the robustness of the optimization results, predictive uncertainty from the three surrogate models (pure-component CMC, β, and SAScore) was propagated through the Rubingh RST framework using a Monte Carlo procedure based on 95% split-conformal prediction intervals. The resulting uncertainty-aware Pareto front is shown in Figure 6, where horizontal error bars represent the propagated 95% prediction intervals of the predicted mixture CMC values. The Pareto front was recomputed across the Monte Carlo ensemble, and the robustness of each candidate was quantified by P­(Pareto), defined as the probability that a formulation remains non-dominated under the propagated uncertainty model. The analysis indicates that no single composition point can be identified as a statistically unique optimum; however, the SDS/CTAB (anionic–cationic) family consistently dominates the Pareto front across the ensemble, with a cumulative P­(Pareto) = 0.73 for the leading compositions. The optimization result is therefore more appropriately interpreted as a robust chemical-family and composition-range recommendation (*x*_SDS_ between 0.10 and 0.40) rather than as a single optimal formulation.

**Figure 6:**
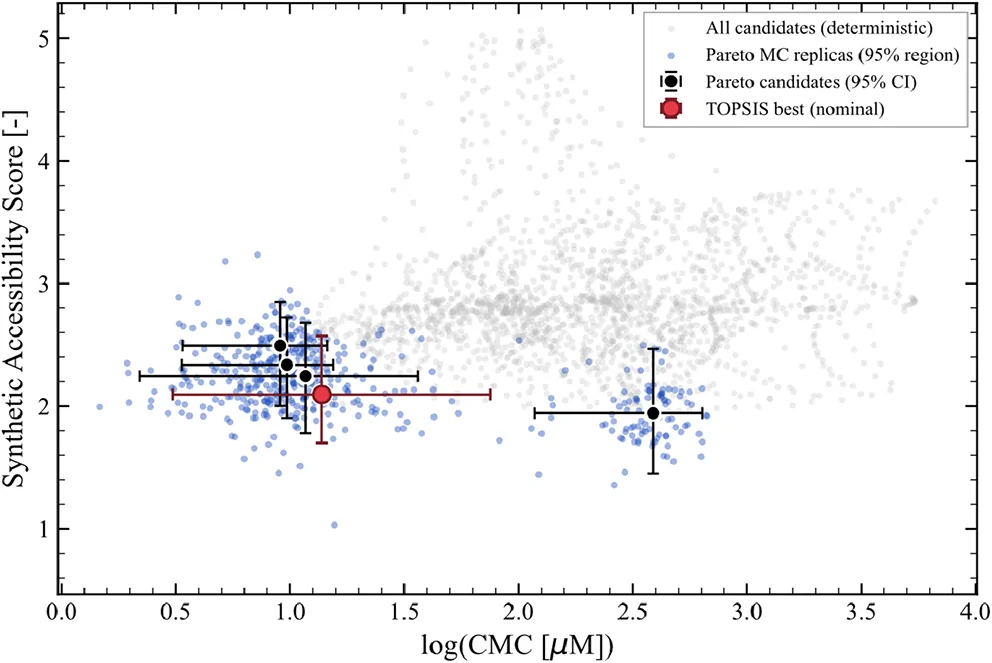
Uncertainty-aware Pareto front obtained from the multi-objective optimisation of binary surfactant blends on the log­(CMC [μM]) vs SAScore. Grey points show all evaluated candidates, the blue point cloud shows the 100 Monte Carlo replicas of the Pareto candidates obtained by jointly propagating the 95 % split-conformal residuals of the β, CMC and SAScore surrogate models. Black markers with error bars indicate the Pareto-optimal (non-dominated) set with their 95 % conformal predictive intervals on both objectives, and the red marker highlights the TOPSIS-selected candidate.

The results obtained are consistent with the underlying dataset used for the development of the β-interaction model, in which the SDS–CTAB best performing system is explicitly included within the training space, and ranks favorably in terms of SAScore. compositions, in excellent agreement with the predicted CMC for these strongly synergistic catanionic systems. Moreover, the CTAB–palmitoyl isethionate pair emerges as one of the most promising Pareto-optimal systems and, importantly, lies outside the binary training space used to fit the β-interaction model, representing a genuine out-of-training prediction of the proposed framework. The uncertainty-aware optimization places this pair on the Pareto front at cationic-rich compositions (*x*_CTAB_ between 0.4 and 0.5), with a strongly synergistic predicted interaction parameter (β = −11.5) and a predicted log­(CMC [μM]) between 0.96 and 0.99. This formulation is ranked immediately after the two SDS–CTAB solutions by TOPSIS (positions 3 and 4). To the best of our knowledge, no experimental mixed-CMC data are available for this binary pair, preventing a direct quantitative comparison. Nevertheless, the predicted behaviour is expected to exhibit strong electrostatic attraction and non-ideal mixed-micelle formation. We therefore identify CTAB–palmitoyl isethionate as a particularly attractive target for future experimental validation.

## 4 Conclusions

The framework proposed provides a robust and reliable representation of surfactant mixtures while significantly reducing the experimental effort required for their characterization. It enables rational, data-driven formulation design, thereby accelerating product development and supporting the design of more efficient and sustainable surfactant systems reducing the amount of surfactant needed. Compared to the current state-of-the-art approaches for estimating mixture surfactant properties, this framework achieves competitive predictive performance while providing thermodynamic informed interpretation of mixture behavior. Critically, the interaction parameter β is not merely an indicator of synergistic or antagonistic deviations in CMC. Within Rubingh’s RST, it governs the non-ideal thermodynamics of mixed micellization, determining the shape of the CMC–composition profile, as well as the existence and position of CMC minima and the equilibrium micellar composition. These interaction parameters directly influence the equilibrium concentration of freely dissolved monomers of each surfactant species below the CMC. Given that monomer concentrations are closely linked to key performance-relevant phenomena, including ecotoxicological availability and dynamic interfacial behavior, a structure-based model capable of predicting β provides indirect access to a broader spectrum of formulation properties beyond CMC alone. This interpretation naturally enables an extensible modeling paradigm. Because β is predicted as a composition-dependent interaction parameter, β­(*x*), it can be incorporated as an intermediate descriptor within broader machine learning frameworks targeting multiple formulation properties encapsulates fundamental non-ideal interactions in mixed surfactant systems, it can be further incorporated as an intermediate descriptor within broader machine learning frameworks that target multiple formulation properties. In this context, β can also be used as a descriptor for the surface tension at the CMC in binary surfactant mixtures. This highlights the potential of β as an effective descriptor of non-ideal interactions rather than a task-specific fitting parameter and supports the development of unified frameworks that couple thermodynamic theory with machine learning for multi-objective formulation design.

Importantly, the suitability of the proposed framework has been further demonstrated in ternary surfactant mixtures, where the complexity of non-ideal interactions increases substantially. This is particularly relevant given that studies on multicomponent surfactant systems remain scarce, and predictive models for such systems are even more limited. The ability of the framework to maintain consistency and interpretability in these higher-order mixtures highlights its potential as a generalizable tool for complex formulation design. Additionally, the case study illustrates the practical applicability of the approach by demonstrating its ability to capture composition-dependent trends and support recommendations at the chemical-family level. This confirms its usefulness not only as a predictive tool but also as a decision-support framework for guiding formulation screening and optimization.

Finally, the proposed framework provides a foundation for several future developments. In particular, extending the methodology to account explicitly for temperature, pH, ionic strength, and water hardness would broaden its domain of applicability and further enhance its predictive capability and relevance for industrial surfactant formulation design. In addition, future developments may incorporate additional target properties within the multi-objective optimization setting, enabling simultaneous optimization of more properties related with performance and sustainability. This would further strengthen the role of the proposed approach as a unified framework for data-driven and thermodynamically consistent surfactant mixture design.

## Supplementary Material



## Data Availability

Data, results and models related to this article can be found on the Github repository https://github.com/SofiaGNu/Optimized_Formulation_Design_Surfactant_Blends and are available under a CC BY 4.0 license.

## Notes

Best-performing hyperparameter configurations of the ANN, LGB, and XGB β-regressors obtained by Bayesian optimization (Tables S1–S3); parity plots of predicted versus experimentally derived β values under the random-composition and unseen-pair validation splits for the LGB regressor (Figure S1) and the XGB regressor (Figure S2); experimental and predicted CMC as a function of surfactant mole fraction for held-out binary pairs (Figure S3); end-to-end parity plot of predicted versus experimental binary-mixture CMC values under the ideal-mixing assumption (β = 0) (Figure S4); comparative performance metrics for end-to-end CMC prediction using β = 0 and ANN-predicted β on the same held-out dataset (Table S4); and the surfactant library used in the optimization case study, including SMILES strings, head-group charge class, and common names (Table S5) (PDF)

## References

[1] Rosen, M. J. ; Kunjappu, J. T. Surfactants and Interfacial Phenomena; John Wiley & Sons, 2012.

[2] Kralova I., Sjöblom J. (2009). Surfactants
Used in Food Industry: A Review. J. Dispersion
Sci. Technol..

[3] Negin C., Ali S., Xie Q. (2017). Most Common Surfactants
Employed in Chemical Enhanced
Oil Recovery. Petroleum.

[4] Panda A., Kumar A., Mishra S., Mohapatra S. S. (2020). Soapnut:
A Replacement of Synthetic Surfactant for Cosmetic and Biomedical
Applications. Sustainable Chem. Pharm..

[5] Grady B. P. (2023). Surfactant
Mixtures: A Short Review. J. Surfactants Deterg..

[6] Lim Y. S., Baharudin N. B., Ung Y. W. (2019). Methyl Ester Sulfonate: A High-performance
Surfactant Capable of Reducing Builders Dosage in Detergents. J. Surfactants Deterg..

[7] Rocha B., de Morais L. A., Viana M. C., Carneiro G. (2023). Promising Strategies
for Improving Oral Bioavailability of Poor Water-Soluble Drugs. Expert Opin. Drug Discovery.

[8] Bureiko A., Trybala A., Kovalchuk N., Starov V. (2015). Current Applications
of Foams Formed from Mixed Surfactant–Polymer Solutions. Adv. Colloid Interface Sci..

[9] Pal N., Vajpayee M., Mandal A. (2019). Cationic/Nonionic
Mixed Surfactants
as Enhanced Oil Recovery Fluids: Influence of Mixed Micellization
and Polymer Association on Interfacial, Rheological, and Rock-Wetting
Characteristics. Energy Fuels.

[10] Zhang R., Somasundaran P. (2006). Advances in Adsorption of Surfactants and Their Mixtures
at Solid/Solution Interfaces. Adv. Colloid Interface
Sci..

[11] Nagtode V. S., Cardoza C., Yasin H. K. A., Mali S. N., Tambe S. M., Roy P., Singh K., Goel A., Amin P. D., Thorat B. R., Cruz J. N., Pratap A. P. (2023). Green Surfactants
(Biosurfactants):
A Petroleum-Free Substitute for Sustainability—Comparison,
Applications, Market, and Future Prospects. ACS Omega.

[12] Brozos C., Rittig J. G., Bhattacharya S., Akanny E., Kohlmann C., Mitsos A. (2024). Graph Neural Networks
for Surfactant Multi-Property
Prediction. Colloids Surf., A.

[13] Motomura K., Yamanaka M., Aratono M. (1984). Thermodynamic
Consideration of the
Mixed Micelle of Surfactants. Colloid Polym.
Sci..

[14] Zhou Q., Rosen M. J. (2003). Molecular Interactions of Surfactants in Mixed Monolayers
at the Air/Aqueous Solution Interface and in Mixed Micelles in Aqueous
Media: The Regular Solution Approach. Langmuir.

[15] Holland P.
M., Rubingh D. N. (1983). Nonideal
Multicomponent Mixed Micelle Model. J. Phys.
Chem. A.

[16] Sarmoria C., Puvvada S., Blankschtein D. (1992). Prediction of Critical Micelle Concentrations
of Nonideal Binary Surfactant Mixtures. Langmuir.

[17] Maeda H. (2005). A Thermodynamic
Analysis of Charged Mixed Micelles in Water. J. Phys. Chem. B.

[18] Rosen M. J., Zhao F. (1983). Binary Mixtures of
Surfactants. The Effect of Structural and Microenvironmental
Factors on Molecular Interaction at the Aqueous Solution/Air Interface. J. Colloid Interface Sci..

[19] Shah S. K., Chakraborty G., Bhattarai A., De R. (2022). Synergistic and Antagonistic
Effects in Micellization of Mixed Surfactants. J. Mol. Liq..

[20] Klima K. M., Koh C. H., Brouwers H. J. H., Yu Q. (2022). Synergistic
Effect
of Surfactants in Porous Geopolymer: Tailoring Pore Size and Pore
Connectivity. Cem. Concr. Compos..

[21] Parra J. G., Iza P., Dominguez H., Schott E., Zarate X. (2020). Effect of Triton X-100
Surfactant on the Interfacial Activity of Ionic Surfactants SDS, CTAB
and SDBS at the Air/Water Interface: A Study Using Molecular Dynamic
Simulations. Colloids Surf., A.

[22] Owoyomi O., Jide I., Akanni M. S., Soriyan O. O., Morakinyo M. K. (2005). Interactions
between Sodium Dodecylsulphate and Triton X-100: Molecular Properties
and Kinetics Investigations. J. Appl. Sci..

[23] Ruiz C. C., Aguiar J. (2000). Interaction, Stability, and Microenvironmental
Properties
of Mixed Micelles of Triton X100 and n-Alkyltrimethylammonium Bromides:
Influence of Alkyl Chain Length. Langmuir.

[24] Ćirin D. M., Poša M. M., Krstonošić V. S. (2012). Interactions between
Sodium Cholate or Sodium Deoxycholate and Nonionic Surfactant (Tween
20 or Tween 60) in Aqueous Solution. Ind. Eng.
Chem. Res..

[25] Zhou Y., Jin Y., Shen Y., Shi L., Lai S., Tang Y. (2021). Strong Synergistic
Effect of Cationic Hydrocarbon Surfactant and Novel Nonionic Tri-Block
Short-Chain Fluorocarbon Surfactant Mixtures on Surface Activity,
Wettability and Solubilization. J. Leather Sci.
Eng..

[26] Alargova R. G., Kochijashky I. I., Sierra M. L., Kwetkat K., Zana R. (2001). Mixed Micellization
of Dimeric (Gemini) Surfactants and Conventional Surfactants: II.
CMC and Micelle Aggregation Numbers for Various Mixtures. J. Colloid Interface Sci..

[27] Kume G., Gallotti M., Nunes G. (2008). Review on
Anionic/Cationic Surfactant
Mixtures. J. Surfactants Deterg..

[28] Phaodee P., Sabatini D. A. (2021). Anionic and Cationic
Surfactant Synergism: Minimizing
Precipitation, Microemulsion Formation, and Enhanced Solubilization
and Surface Modification. J. Surfactants Deterg..

[29] Li J., Peng M., Yang Y., Zhu H., Zhang D., Jiang B. (2025). Surface Activity, Aggregation Behavior,
and Wettability of a Mixture
of Perfluorinated Branched Short Chain Sulfonate Surfactants and Hydrocarbon
Cationic Surfactants in Dilute Solutions. Langmuir.

[30] Ma K., Li P., Wang Z., Chen Y., Campana M., Doutch J., Dalgliesh R., Maestro A., Thomas R. K., Penfold J. (2022). Strong Synergistic
Interactions in Zwitterionic–Anionic Surfactant Mixtures at
the Air–Water Interface and in Micelles: The Role of Steric
and Electrostatic Interactions. J. Colloid Interface
Sci..

[31] Mohamad-Aziz S. N., Mishra P., Zularisam A. W., Sakinah A. M. M. (2019). Isooctane-Based
Anionic and Zwitterionic Surfactant: Synergistic Interaction of Mixed
Reverse Micelle and Solubilisation of Erythromycin. J. Mol. Liq..

[32] Choudhary A., Desai S., Kamruzzaman M., Landera A., Ghosh K., Poorey K. (2025). Predicting the Critical
Micelle Concentration of Binary
Surfactant Mixtures Using Machine Learning. J. Cheminf..

[33] Qing D., Lu Z., Hou L., Dai W., Hong J., Ge X. (2025). Predicting
the Surface Tension of Mixed Surfactant Systems through Theory and
Machine Learning. Colloids Surf., A.

[34] Brozos C., Rittig J. G., Akanny E., Bhattacharya S., Kohlmann C., Mitsos A. (2025). Predicting the Temperature-Dependent
CMC of Surfactant Mixtures with Graph Neural Networks. Comput. Chem. Eng..

[35] Han W., Tan J., Peng L., Liu L., Zhou X., Zhang W., Shi B. (2019). Ecotoxicity and Micellization
Behavior of Anionic Surfactant Sodium
Dodecylbenzene Sulfonate (SDBS) and Its Mixtures with Nonionic Surfactant
Fatty Alcohol-Polyoxyethylene Ether (AEO). Aquat.
Toxicol..

[36] Virtanen P., Gommers R., Oliphant T. E., Haberland M., Reddy T., Cournapeau D., Burovski E., Peterson P., Weckesser W., Bright J. (2020). SciPy 1.0: Fundamental
Algorithms for Scientific Computing in Python. Nat. Methods.

[37] Nocedal, J. ; Wright, S. J. Numerical Optimization; Springer, 2006.

[38] Moriwaki H., Tian Y.-S., Kawashita N., Takagi T. (2018). Mordred: A Molecular
Descriptor Calculator. J. Cheminf..

[39] Mavrotas G. (2009). Effective
Implementation of the ε-Constraint Method in Multi-Objective
Mathematical Programming Problems. Appl. Math.
Comput..

[40] Ray G. B., Chakraborty I., Ghosh S., Moulik S. P., Holgate C., Glenn K., Palepu R. M. (2007). Studies on Binary
and Ternary Amphiphile
Combinations of Tetradecyltrimethylammonium Bromide (C14TAB), Tetradecyltriphenylphosphonium
Bromide (C14TPB), and Tetradecylpyridinium Bromide (C14PB). A Critical
Analysis of Their Interfacial and Bulk Behaviors. J. Phys. Chem. B.

[41] Dar A. A., Rather G. M., Das A. R. (2007). Mixed Micelle Formation and Solubilization
Behavior toward Polycyclic Aromatic Hydrocarbons of Binary and Ternary
Cationic–Nonionic Surfactant Mixtures. J. Phys. Chem. B.

[42] Graciaa A., Ghoulam M. B., Marion G., Lachaise J. (1989). Critical Concentrations
and Compositions of Mixed Micelles of Sodium Dodecylbenzenesulfonate,
Tetradecyltrimethylammonium Bromide and Polyoxyethylene Octylphenols. J. Phys. Chem. A.

[43] Murphy A., Taggart G. (2002). A Comparison of Predicted
and Experimental Critical
Micelle Concentration Values of Cationic and Anionic Ternary Surfactant
Mixtures Using Molecular-Thermodynamic Theory and Pseudophase Separation
Theory. Colloids Surf., A.

[44] González-Núñez S., Sumer Z., Amador C., Madhav P., Muralidharan A., Adjiman C. S., Martín M. (2025). Design of Surfactant Molecules Under
Performance Constraints. ACS Sustainable Chem.
Eng..

[45] Qin S., Jin T., Van Lehn R. C., Zavala V. M. (2021). Predicting Critical
Micelle Concentrations
for Surfactants Using Graph Convolutional Neural Networks. J. Phys. Chem. B.

[46] Seddon D., Müller E. A., Cabral J. T. (2022). Machine Learning Hybrid Approach
for the Prediction of Surface Tension Profiles of Hydrocarbon Surfactants
in Aqueous Solution. J. Colloid Interface Sci..

[47] Ertl P., Schuffenhauer A. (2009). Estimation
of Synthetic Accessibility Score of Drug-like
Molecules Based on Molecular Complexity and Fragment Contributions. J. Cheminf..

[48] Azum N., Rub M. A., Asiri A. M. (2016). Micellization
and Interfacial Behavior
of Binary and Ternary Mixtures in Aqueous Medium. J. Mol. Liq..

[49] Ghosh S. (2001). Surface Chemical
and Micellar Properties of Binary and Ternary Surfactant Mixtures
(Cetyl Pyridinium Chloride, Tween-40, and Brij-56) in an Aqueous Medium. J. Colloid Interface Sci..

[50] Dar A. A., Rather G. M., Ghosh S., Das A. R. (2008). Micellization and
Interfacial Behavior of Binary and Ternary Mixtures of Model Cationic
and Nonionic Surfactants in Aqueous NaCl Medium. J. Colloid Interface Sci..

[51] Zhang Q. H., Sheng R., Ren Z. H., Huang J., Wang Y. X., Wang B. R., Huang X. L., Cheng Q., Wu X. M., Wang T. B. (2023). Interaction and
Micellar Behavior of Ternary Mixture
of Amphoteric Amino Sulfonate Surfactant with Traditional Anionic
and Nonionic Surfactants: Effect of Hydrophilicity. J. Ind. Eng. Chem..

[52] Wang Z., Rangaiah G. P. (2017). Application and Analysis of Methods
for Selecting an
Optimal Solution from the Pareto-Optimal Front Obtained by Multiobjective
Optimization. Ind. Eng. Chem. Res..

