# Supplementary material for: Predicting Synergistic and Antagonistic Micellization Effects in Surfactant Blends for Optimized Formulation Design
Source: J Chem Inf Model. 2026 Sep 29;66(19):12463–76. doi: 10.1021/acs.jcim.6c02030 (PMC13663854; doi:10.1021/acs.jcim.6c02030)
Supplement: Supplementary file 1 [file ci6c02030_si_001.pdf]

Predicting Synergistic and Antagonistic Micellization Effects in Surfactant Blends for Optimized Formulation Design

Sofía González-Núñez[a], Carlos Amador[b], Mariano Martín[a*1]

[a] Departamento de Ingeniería Química. Universidad de Salamanca. Pza. Caídos 1-5, 37008 Salamanca, Spain
[b] Newcastle Innovative Centre. Procter and Gamble. Whitley Rd, Longbenton, Newcastle Upon Tyne, Tyne And Wear NE12 9SR, England

This document provides additional methodological detail and diagnostic results supporting the main manuscript. Tables S1–S3 report the best-performing hyperparameter configurations of the ANN, LGB and XGB β-regressors obtained by Bayesian optimization. Figures S1 and S2 show parity plots of predicted versus experimentally derived β values for the LGB and XGB regressors under the random-composition and unseen-pair splits, complementing the ANN parity plot in the main text. Figure S3 compares experimental and predicted CMC as a function of surfactant mole fraction for all held-out binary pairs with at least four measured composition points, and Figure S4 shows the corresponding end-to-end held-out parity plot obtained under the ideal-mixing assumption $\beta = 0$. Table S4 quantifies the improvement obtained by replacing $\beta = 0$ with the ANN-predicted β on the same held-out set, and Table S5 lists the surfactant library used in the case study.

[1] M. Martín mariano.m3@usal.es

Feed-forward neural network (ANN)

Implemented as sklearn.neural_network.MLPRegressor, wrapped in a standard scikit-learn Pipeline with a median imputer, near-zero-variance filter and a StandardScaler before the network.

Table S1. Hyperparameters of the feed-forward neural network (ANN) regressor used for the β interaction parameter.

Hyperparameter	Value
Hidden layer sizes	(256, 128, 64, 32)
Activation	ReLU
Solver	Adam
L2 regularisation α	1.187×10^{-3}
Learning-rate schedule	Adaptive
Initial learning rate	4.022×10^{-4}
Maximum epochs	300
Early stopping	Enabled
Validation fraction	0.10
Patience (n_iter_no_change)	50
Random seed	42

Gradient-boosted trees, LightGBM (LGB)

Implemented as lightgbm.LGBMRegressor. No feature scaling is applied (scale-invariant model).

Table S2. Hyperparameters of the LightGBM gradient-boosted trees (LGB) regressor used for the β interaction parameter.

Hyperparameter	Value
Objective	regression (L2)
n_estimators	800
num_leaves	31
learning_rate	0.01
L1 regularisation (lambda_l1)	1.0
L2 regularisation (lambda_l2)	8.0
Row subsample per tree (subsample)	0.75
Feature subsample per tree (colsample_bytree)	0.75
Random seed	42

Gradient-boosted trees, XGBoost (XGB)

Implemented as xgboost.XGBRegressor with the histogram tree method. No feature scaling is applied.

Table S3. Hyperparameters of the XGBoost gradient-boosted trees (XGB) regressor used for the β interaction parameter.

Hyperparameter	Value
Objective	reg:squarederror
Tree method	hist
n_estimators	1 396
learning_rate	4.532×10^{-2}
max_depth	4
min_child_weight	10

Row subsample per tree (subsample)	0.90
Feature subsample per tree (colsample_bytree)	0.50
Minimum split loss (gamma)	0.892
L1 regularisation (reg_alpha)	0.30
L2 regularisation (reg_lambda)	16.0
Random seed	42

The parity plots for the LGB and XGB models are provided below for comparison. Since the ANN model was selected as the final predictive model used throughout this study, its parity plot is presented in the main manuscript.

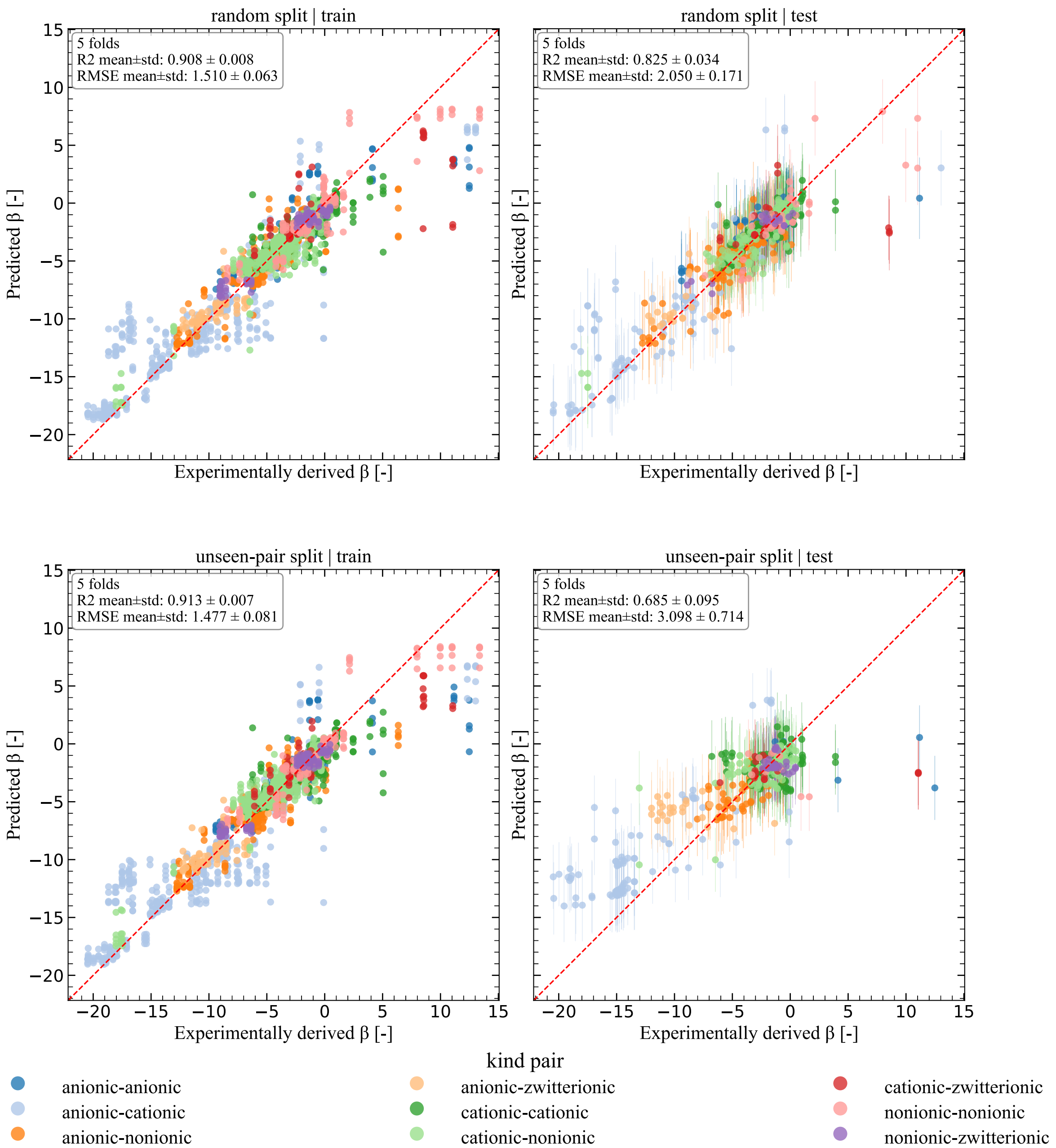

Figure S1. Parity plots of predicted versus experimentally derived β values for the LGB model under two data-splitting strategies. Rows correspond to the random composition split (80/20 split of individual observations; top) and the unseen-pair split (80/20 split of unique binary surfactant pairs; bottom), while columns show predictions for the training (left) and test (right) partitions. Points are coloured according to the surfactant charge-class

combination (see legend). The dashed red line denotes the ideal y = x relationship. Vertical bars in the test panels represent 95% split-conformal prediction intervals.

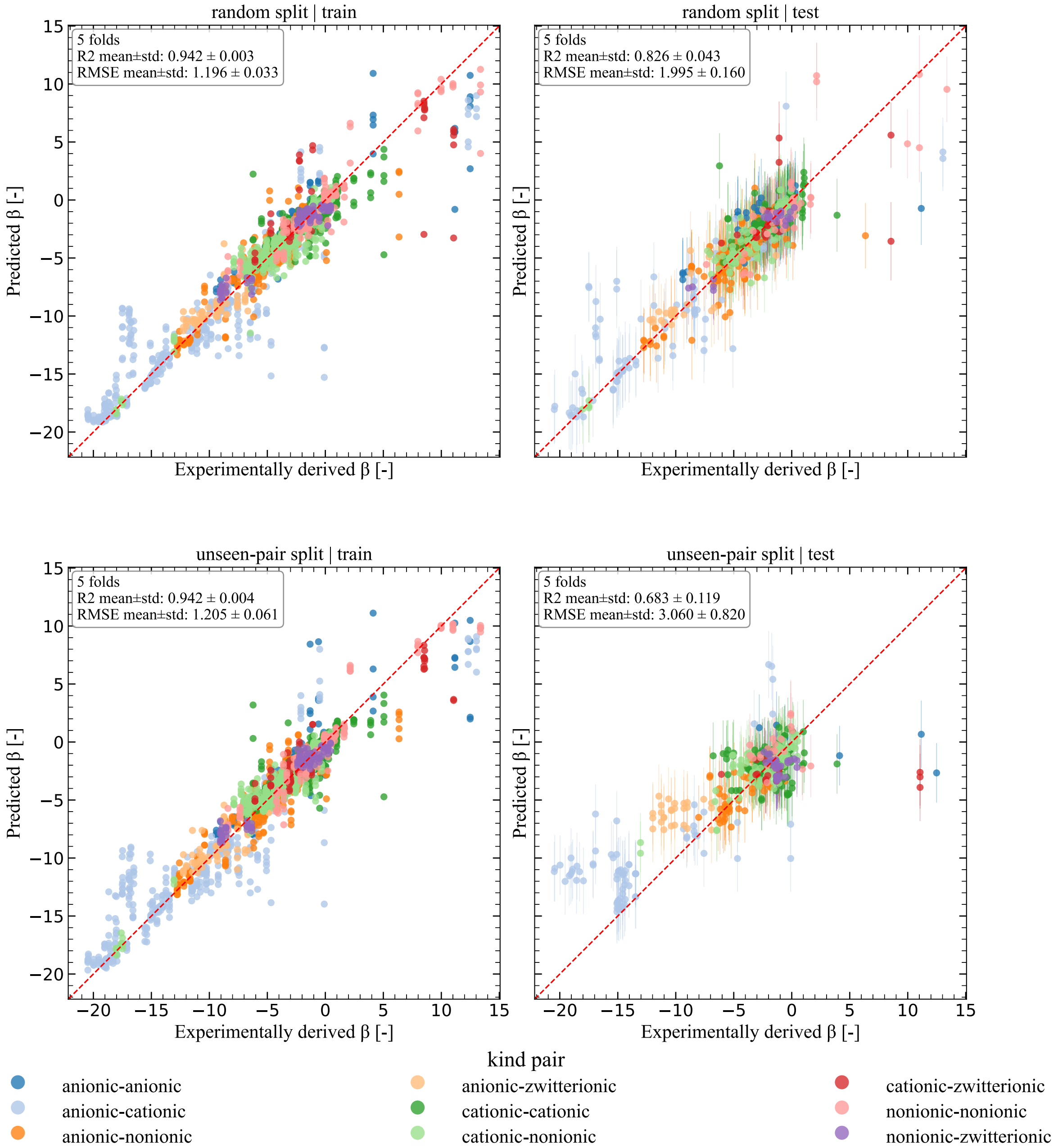

Figure S2. Parity plots of predicted versus experimentally derived β values for the XGB model under two data-splitting strategies. Rows correspond to the random composition split (80/20 split of individual observations; top) and the unseen-pair split (80/20 split of unique binary surfactant pairs; bottom), while columns show predictions for the training (left) and test (right) partitions. Points are coloured according to the surfactant charge-class combination (see legend). The dashed red line denotes the ideal y = x relationship. Vertical bars in the test panels represent 95% split-conformal prediction intervals.

To evaluate whether predictive uncertainty could lead to systematic displacement of the composition at which the mixed CMC reaches its minimum, we compared the experimental and predicted CMC profiles for every held-out

binary pair containing at least four measured composition points. The resulting panel-by-panel comparison (Figure S3) shows that the proposed framework reproduces the overall shape of the CMC-composition relationship and captures the location of the CMC minimum with good agreement across most systems. In particular, no systematic shift of the minimum is observed for the charge-pair classes that dominate the strongly synergistic region of the dataset, supporting the use of the framework for identifying composition ranges associated with enhanced mixed-micelle formation.

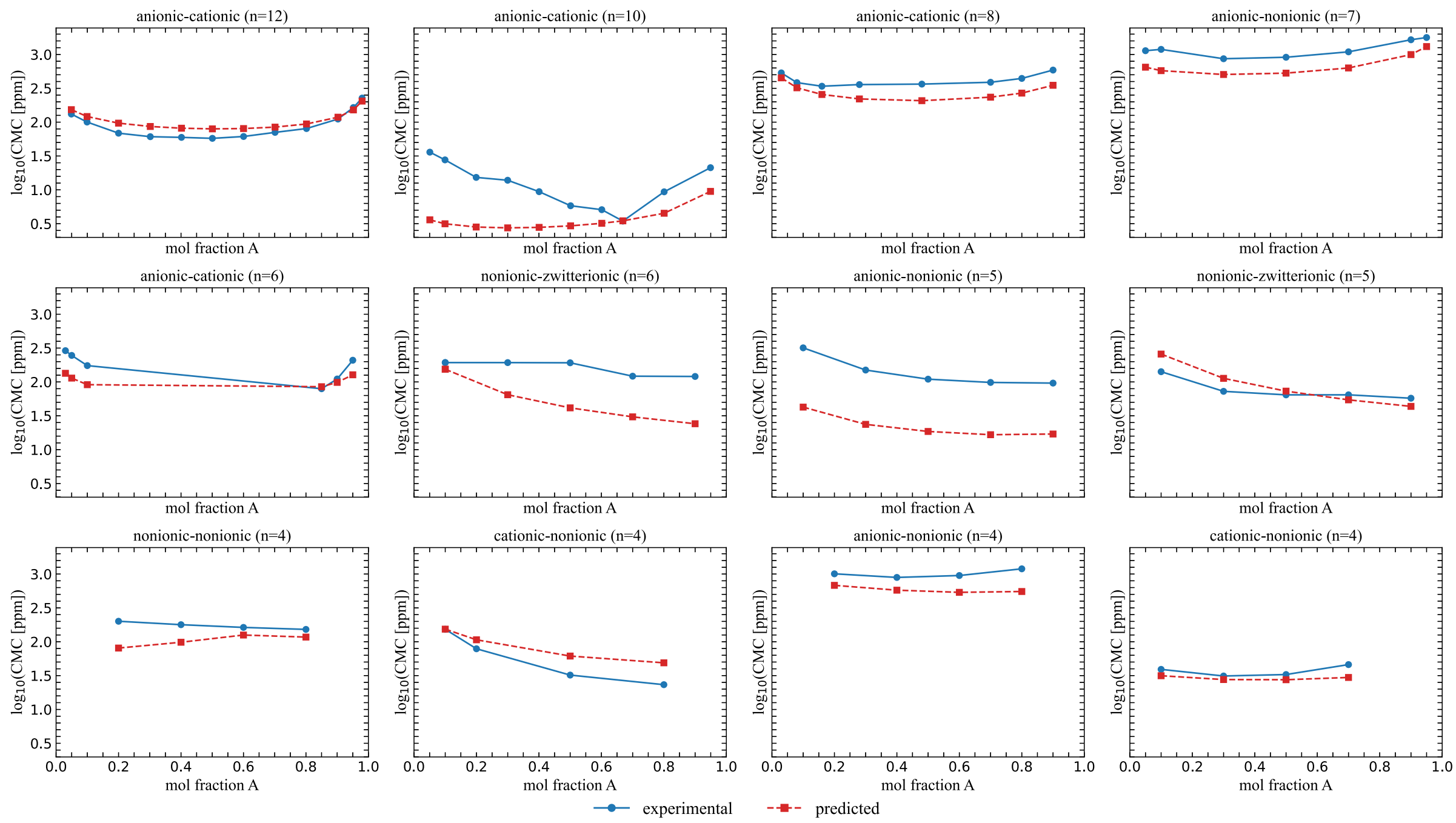

Figure S3. Experimental and predicted CMC as a function of surfactant mole fraction for all held-out binary surfactant pairs containing at least four experimental composition points (12 pairs in total).

To quantify the predictive contribution of the interaction parameter, the end-to-end validation described in Section 3.3 was repeated under the ideal-mixing assumption ($\beta = 0$). The comparison was performed using the same 96 held-out binary mixtures, for which the corresponding surfactant pairs were completely excluded from the training set of the β model under the unseen-pair split strategy. In both cases, the pure-component CMC model, mixture compositions, and Rubingh RST solver were kept identical; only the interaction parameter differed, allowing the effect of β to be isolated directly. As shown in Table S4 and Figure S4, the hybrid route reduces the log-CMC RMSE by 41% (0.587 to 0.349) and lifts R^2 from 0.25 to 0.77 relative to the ideal-mixing benchmark on the same pairs. Since the pure-component CMCs, mole fractions and RST solver are identical in both rows, this improvement is unambiguously attributable to the ANN-predicted β interaction term. The gain is largest for the two

dominant charge classes on the split (anionic-cationic and cationic-nonionic), which are precisely the classes for which surfactant synergy is thermodynamically expected and where β differs most from zero. For classes where β naturally sits near zero on this split (nonionic-nonionic, some nonionic-zwitterionic mixtures) the two routes yield comparable log-CMC errors, again in line with the physical role of β.

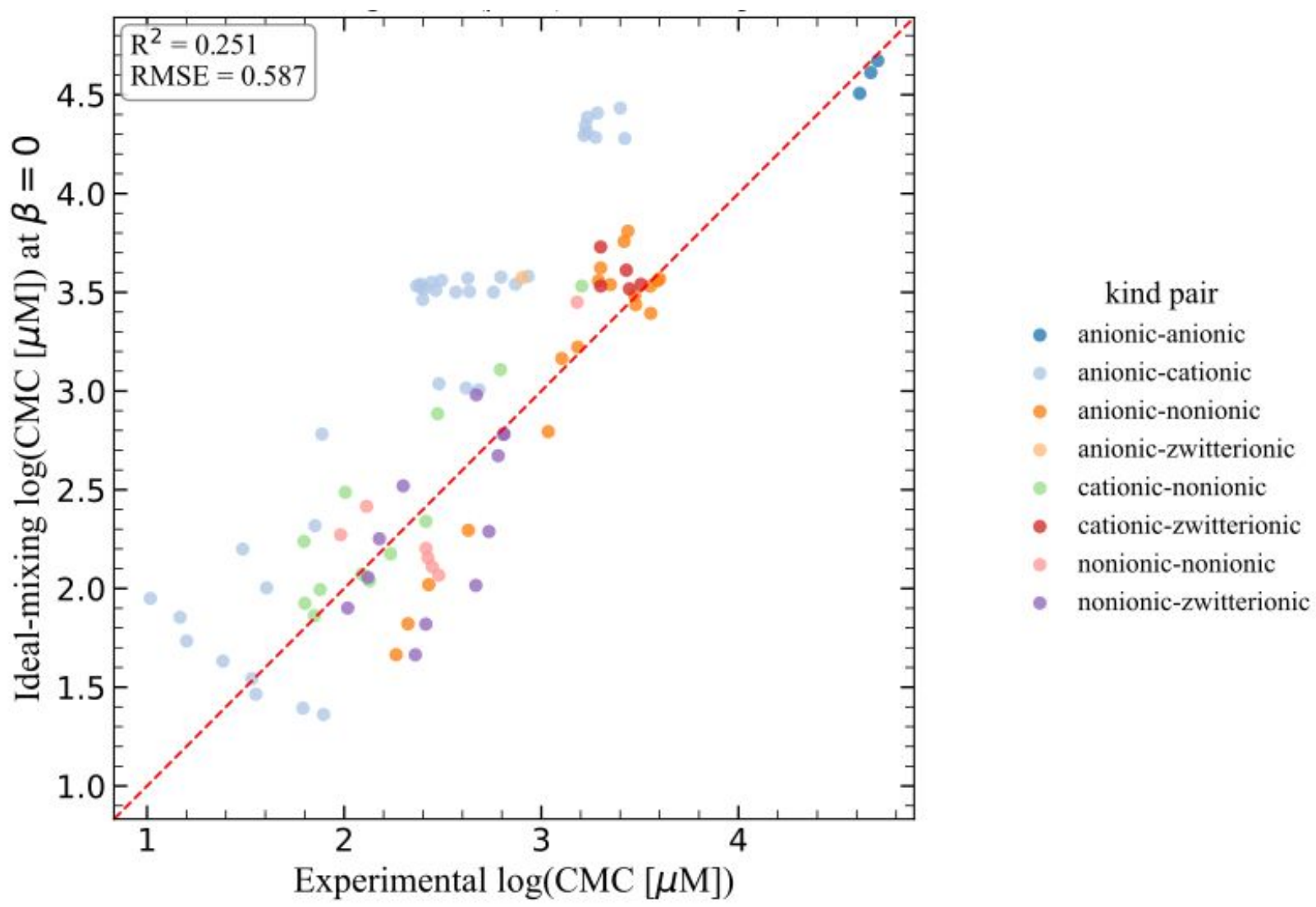

Figure S4. End-to-end held-out parity plot of predicted versus experimental log(CMC [ppm]) for binary surfactant mixtures under the ideal-mixing assumption (β = 0). Each point represents a binary mixture whose surfactant pair was completely excluded from the training set of the β model under the unseen-pair split strategy. Points are color-coded according to the surfactant charge-pair class.

Table S4. Comparison of end-to-end binary-mixture CMC prediction performance on the same held-out evaluation set (surfactant pairs excluded from β-model training under the unseen-pair split strategy) using the ideal-mixing assumption (β = 0) and the ANN-predicted interaction parameter β.

	R^2	RMSE	MAE
**Ideal mixing (β = 0)**	0.251	0.587	0.456
**ANN β**	0.768	0.349	0.265

The surfactant library used in the proposed case study spans a broad range of molecular architectures and interfacial chemistries The dataset includes classical linear surfactants, aromatic alkylbenzene derivatives, polyoxyethylene-based non-ionic surfactants, sugar-based amphiphiles, and hybrid structures combining multiple functional motifs, as reported in Table S5. This diversity enables the model to learn across fundamentally different packing regimes and interfacial interaction mechanisms, ranging from purely electrostatic to strongly sterically constrained systems.

Table S5. Surfactant set used for case study evaluation.

SMILES	Charge	Name
**CCCCCCCCCCCCOS(=O)(=O)[O-]**	A	lauryl sulfate (SLS/SDS-type alkyl sulfate)

**CCCCCCCCCCCCc1ccccc1S(=O)(=O)[O-]**	A	Linear alkylbenzene sulfonate (LAS)
**CCCCCCCCCCCCOCCOCCOCCOCCOCCOP(=O)(O)[O-]**	A	Laureth phosphate / ethoxylated alkyl phosphate
**CCCCCCCCCCCCC[N+](C)(C)Cc1ccccc1**	C	Benzalkonium-type quaternary ammonium surfactant
**CCCCCCCCCCOC(=O)CC(C(=O)OCCCCCCCC)S(=O)(=O)[O-]**		Dioctyl sulfosuccinate (AOT-type surfactant)
**CCCCCCCCCCOCCOCCOCCOCCOCCOCCOCCOCCO**	N	C10E8
**CCCCCCCCCCCCOCCOCCOCCOCCOCCOCCOCCOCCO**	N	C12E8
**CCCCCCCCCCCCCCOCCOCCOCCOCCOCCOCCOCCOCCO**	N	C14E8
**CC(C)(C)CC(C)(C)c1ccc(OCCO)cc1**	N	Octylphenol ethoxylate derivative (Triton-type)
**CCCCCCCCOC1O[C](CO)[C](O)[C](O)[C]1O**	N	Octyl glucoside
**CCCCCCCCCCOC1O[C](CO)[C](O)[C](O)[C]1O**	N	Decyl glucoside
**CCCCCCCCCCCC(=O)N(C)CC[N+](C)CC(=O)[O-]**	Z	Amido betaine surfactant (cocamidopropyl betaine-type)
**CCCCCCCCCCCC[N+](C)(C)CC(=O)[O-]**	Z	Lauryl betaine
**CCCCCCCCCCCC[N+](C)(C)CCOCCS(=O)(=O)[O-]**	Z	Sulfobetaine surfactant
**CCCCCCCCCCCC[N+](C)(C)[O-]**	Z	Lauramine oxide
**CCCCCCCCCCCCCCCC[N+](C)(C)(C)**	C	Cetyltrimethylammonium (CTA/CTAB-type)
**CCCCCCCCCCCC[N+](C)(C)(C)**	C	Dodecyltrimethylammonium (DTAB-type)
**CCCCCCCCCCCCCCCC(=O)OCCS(=O)(=O)[O-]**	A	Palmitoyl isethionate
**CCCCCCCCCCCC(=O)NCC(O)C(O)C(O)CO**	N	Lauramide MEA / fatty acid alkanolamide derivative
**CCCCCCCCCCO[C]1O[C](CO)[C](O[C]2O[C](CO)[C](O)[C](O)[C]2O)[C](O)[C]1O**	N	Decyl maltoside
